# Particularly strong immune response to influenza vaccination in patients with decompensated liver cirrhosis linked to systemic inflammation

**DOI:** 10.3389/fimmu.2026.1734093

**Published:** 2026-04-22

**Authors:** Janyn Heisig, Valerie Ohlendorf, Nhan Nguyen, Peggy Riese, Stephanie Trittel, Liang Zhou, Ahmed Alaswad, Heiner Wedemeyer, Anke Kraft, Yang Li, Markus Cornberg, Carlos A. Guzmán, Benjamin Maasoumy

**Affiliations:** 1 Department of Vaccinology and Applied Microbiology, Helmholtz Centre for Infection Research (HZI), Braunschweig, Germany; 2 Department of Gastroenterology, Hepatology, Infectious Diseases and Endocrinology, Hannover Medical School (MHH), Hannover, Germany; 3 PRACTIS Clinician Scientist Program, Dean’s Office for Academic Career Development, Hannover Medical School, Hannover, Germany; 4 Centre for Individualised Infection Medicine (CiiM), A Joint Venture of Helmholtz Centre for Infection Research and Hannover Medical School, Hannover, Germany; 5 TWINCORE, A Joint Venture Between the Helmholtz-Centre for Infection Research and the Hannover Medical School, Hannover, Germany; 6 German Centre for Infection Research (DZIF), Partner Site Hannover-Braunschweig, Braunschweig, Germany; 7 Cluster of Excellence RESIST (EXC 2155), Hannover Medical School, Hannover, Germany; 8 Department of Internal Medicine and Radboud Center for Infectious Diseases, Radboud University Medical Center, Nijmegen, Netherlands

**Keywords:** compensated and decompensated cirrhosis, humoral- and cellular immunity, influenza vaccine, liver cirrhosis, liver disease

## Abstract

**Background and aims:**

Seasonal influenza virus infections represent a global health threat, especially in high-risk groups, including patients with liver cirrhosis that are considered to be immunocompromised, in particular in decompensated stages. Although vaccination is the most cost-efficient tool to prevent infectious diseases, information about vaccine performance in these patients is scarce. This study aimed to dissect the immunological responses to seasonal influenza vaccines in patients suffering from compensated or decompensated liver cirrhosis.

**Approach and results:**

Prospective, observational studies during the influenza seasons 2019-2020 (1^st^ season) and 2020-2021 (2^nd^ season) were performed. Participants received the WHO recommended seasonal tetravalent inactivated influenza vaccine. Samples taken before and after vaccination were subjected to in-depth analyses by serology, cytokine immunoprofiling, multi-parametric flow cytometry, and metabolomics. Patients with liver cirrhosis showed stronger vaccine-induced immune responses in comparison to healthy individuals, including hemagglutination-inhibiting and neutralizing antibodies. Furthermore, enhanced cell-mediated immune responses were observed in the cirrhosis patients as compared to healthy subjects after vaccination. Surprisingly, vaccination response was even stronger in more advanced, decompensated stages of liver cirrhosis. Distinct serum cytokine and metabolite profiles associated with systemic inflammation differentiated patients with decompensated from compensated cirrhosis as well as from the healthy individuals and were linked to vaccine response.

**Conclusion:**

Patients with liver cirrhosis can mount an efficient response to seasonal influenza vaccines that is even superior in more advanced stages of cirrhosis. Systemic inflammation caused by liver cirrhosis may contribute to distinct humoral and cellular vaccine responses.

## Introduction

Liver cirrhosis is considered as a systemic disease that affects several other organs including the immune system. It can be sub-classified into a compensated as well as a decompensated stage, which is characterized by specific clinical complications (1). The alterations in the immune system caused by liver cirrhosis are summarized in the term cirrhosis-associated immune dysfunction (CAID) with its two key modules: systemic inflammation and immune deficiency (2). While the systemic inflammatory phenotype is characterized by a permanent activation of immune cells, the immune deficiency state displays alterations leading to *e.g*., reduced phagocytosis or T cell exhaustion. Along with these two phenotypes, CAID increases the susceptibility to infections (2).

Influenza viruses type A and B cause seasonal epidemics of acute respiratory disease with estimated 3–5 million severe cases annually, including 290,000-650,000 deaths worldwide (3). Patients with liver cirrhosis, who are infected with the influenza virus, have a higher risk of hospitalization and infection-related death (4). Next to severe respiratory symptoms, influenza virus infections are described to trigger hepatic decompensations in liver cirrhosis patients and can even cause acute-on-chronic liver failure (ACLF), a hyperinflammatory syndrome which is associated with multi-organ failure and mortality rates of up to 80% within 28 days (5).

The increased hospitalization and mortality rates as well as the limitation of influenza therapeutics to neuraminidase inhibitors underlines the need for stringent vaccination regimes in patients with liver cirrhosis. Despite limited studies addressing vaccine efficacy in these patients, vaccination is indeed strongly recommended for this vulnerable population (6), as indirect evidence suggests that hospital admission rates are reduced by this preventive measure (7). Unfortunately, the seasonal influenza vaccination coverage is still low in this high-risk group (8). However, considering the knowledge about immune alterations in the context of CAID, there is a paucity of quantitative and qualitative evidence for the effectiveness of influenza vaccines in promoting sero-protection in this patient group (7). Clinical studies investigating cellular immune responses after influenza vaccination are also missing. Furthermore, the underlying immunological mechanisms that may lead to distinct immune responses are still not fully understood. There is evidence that baseline immune signatures, such as cytokine levels, may reflect and predict vaccination outcomes, which could in turn provide the rationale to adapt vaccination strategies (9, 10).

To address these gaps of knowledge on vaccine responsiveness and underlying mechanisms in this high-risk group, this study evaluated the humoral and cellular immune responses after vaccination with tetravalent inactivated influenza vaccines in patients with compensated and decompensated cirrhosis as compared to healthy subjects. We also analyzed baseline cytokine and metabolomics signatures to identify predictive biomarkers correlating with vaccination outcomes.

## Materials and methods

### Study cohort and study design

Patients with liver cirrhosis treated at Hannover Medical School and healthy individuals were included between September 2019 and April 2020 (1^st^ season) and September 2020 and April 2021 (2^nd^ season). Inclusion criteria of the study were: medical indication for receiving seasonal influenza vaccination (11), written informed consent, and age from 18–80 years. Exclusion criteria were missing informed consent, contraindications against influenza vaccination, pregnancy, immunosuppression, symptomatic anemia or hemoglobin <7 g/dl.

Influenza vaccination was performed according to the medical indication and prescribing information applying a single administration of the WHO recommended tetravalent influenza vaccine (2019/2020: Vaxigrip tetra A/Brisbane/02/2018(H1N1)pdm09, A/Kansas/14/2017 (H3N2), B/Colorado/06/2017, B/Phuket/3073/2013; 2020/2021: Influsplit tetra A/Guangdong-Maonan/SWL1536/2019(H1N1)pdm09, A/HongKong/2671/2019(H3N2), B/Washington/02/2019, B/Phuket/3073/2013).

Before vaccination (baseline, BL), on day 2-14 (visit 1, V1), day 18-50 (visit 2, V2), and day 47-105 (visit 3, V3) after vaccination serial venous blood sampling were performed for immunological laboratory testing including measurement of HAI titers, MN titers, flow cytometry, cytokine profiling and measurement of circulating metabolites (details of laboratory testings are described in the supplementary material including **Supplementary Figure 1** and **Supplementary Table 1**). Sample numbers for each assay are given in **Supplementary Table 2**. Additionally, at BL routine laboratory parameters were analyzed by the central laboratory of Hannover Medical School, and clinical data of study participants as well as information about previous influenza vaccinations were collected. Cirrhotic patients were staged by the Child Pugh Score (12) in compensated liver cirrhosis (stage A [5–6 points] and decompensated liver cirrhosis (stage B [7–9 points] and C [>9 points]).

### Ethics

All research was conducted in accordance with both the Declarations of Helsinki and Istanbul. The study was approved by the local ethics committee of Hannover Medical School (8614_BO_S_2019). All subjects gave written informed consent for study participation. Study participation had neither an impact on the indication for vaccination, selection of the specific vaccine candidate nor the vaccination regimen. Patients or the public were not involved in the design, or conduct, or reporting, or dissemination plans of our research.

### Statistical analysis

Statistical analyses were performed using the software GraphPad Prism version 9.1.2 or 9.4.1 (GraphPad Software, San Diego, California USA). The Mann-Whitney U test was used for testing independence of continuous variables. Proportions of high- and low-/non-responder were determined, and significance of categorical parameters was analyzed by using the fisher’s exact test. Further comparison between the study groups was done by the two-way ANOVA with the uncorrected Fisher’s LSD test. Correlation analysis was done with the Spearman correlation coefficient r. P-values of ≤0.05 were considered significant.

The metabolic analysis was performed with R version 4.2.0. The metabolite concentrations (General Metabolics) and HAI titers were log2 transformed. A linear model with age and sex as co-factors was used to estimate the association of metabolite concentrations to compensated and decompensated cirrhotic patients compared to healthy individuals. Other linear model with age, sex, and disease conditions as co-factors were used to estimate the association between metabolite concentration at BL and the post-vaccination (V2) HAI titer. P-values of ≤0.05 were considered significant. P-adj was calculated from p-values using the FDR method. The pathway analysis was applied for metabolites that were significantly associated with the post-vaccination (V2) HAI titer in at least two antigens in the 1^st^ season and showed a consistent in the direction of association with HAI titer in at least 75% across antigens and two seasons. The metabolites were annotated to HMDB, KEGG compound, and Chemical Entities of Biological Interest (ChEBI) IDs. The pathway analysis was performed using the Metabo Analyst website (13) using these annotated metabolite IDs. The code for metabolite analysis is accessible at https://github.com/CiiM-Bioinformatics-group/ZirFlu/.

## Results

### BL characteristics of the study population

A number of 41 patients with liver cirrhosis and 69 healthy individuals, distributed in two seasons, were included in the study. In total, 5 cirrhotic patients and 3 healthy individuals were lost to follow-up after vaccination and were therefore excluded from the further analyses.

Details of the study population and differences between the groups in selected BL parameters are given in **Table 1** as well as in **Supplementary Tables 3-7**.

**Table 1:** Differences in BL parameters between healthy individuals and cirrhotic patients (1st season; Mann-Whitney-U Test).

Parameter	Healthy individuals (N; median (min-max)	Cirrhotic patients (N; median(min-max)	P-value
Male sex	17/33 (51.51%)	22/28 (78.57%)	0.0351
Age (years)	33; 40.0 (25.0-65.0)	28; 52.0 (27.0-71)	0.0003
BMI (kg/m2)	13; 22.41 (19.3-27.46)	26; 27.98 (17.2–50.8)	0.0014
White cell count (tsd/µl)	32; 5.9 (4.0-10.2)	28; 5.1 (2.1-9.1)	0.0202
Lymphocytes (tsd/µl)	32; 2.05 (1.09-3.47)	28; 1.285 (0.25-2.65)	<0.0001
Platelets (tsd/µl)	32; 244.5 (192-386)	28; 86 (35-273)	<0.0001
INR	32; 0.89 (0.83-0.99)	28; 1.2 (0.95-1.73)	<0.0001
Creatinine (mmol/l)	32; 77.0 (57.0-108.0)	28; 84.5 (53.0-242.0)	0.0376
Sodium (mmol/l)	32; 140.0 (135.0-144.0)	28; 138.5 (123.0-144.0)	0.1053
Albumin (g/l)	32; 46.50 (41.0-56.0)	28; 38.50 (26.0-51.0)	<0.0001
Bilirubin (mmol/l)	32; 9.0 (3.0-32.0)	28; 17.5 (5-143)	<0.0001
AST (U/l)	32; 24.50 (13.0-53.0)	28; 42 (21.0-168.0)	<0.0001
ALT (U/l)	32; 21.5 (13.0-79.0)	28; 40.5 (13.0-221.0)	0.0020
IgG (g/l)	32; 11.46 (5.26-13.79)	28; 17.53 (7.9-38.5)	<0.0001
IgM (g/l)	32; 0.90 (0.25-1.85)	28; 1.295 (0.3-6.4)	0.0046
HbA1c %	29; 5.10 (4.60-7.40)	28; 5.25 (4.0-11.0)	0.7658
Previous influenza vaccination	22/28 (78.6%)	10/28 (35.7%)	0.0026

BMI, body mass index; INR, international normalized ratio; AST, aspartate aminotransferase; ALT, alanine aminotransferase; IgG, immunoglobulin G; IgM, immunoglobulin M.

Statistically significant differences in BL parameters between healthy individuals and cirrhotic patients are indicated in red.

### Cirrhotic patients display higher levels of antibody titers and neutralizing activities following vaccination

**Figure 1:**
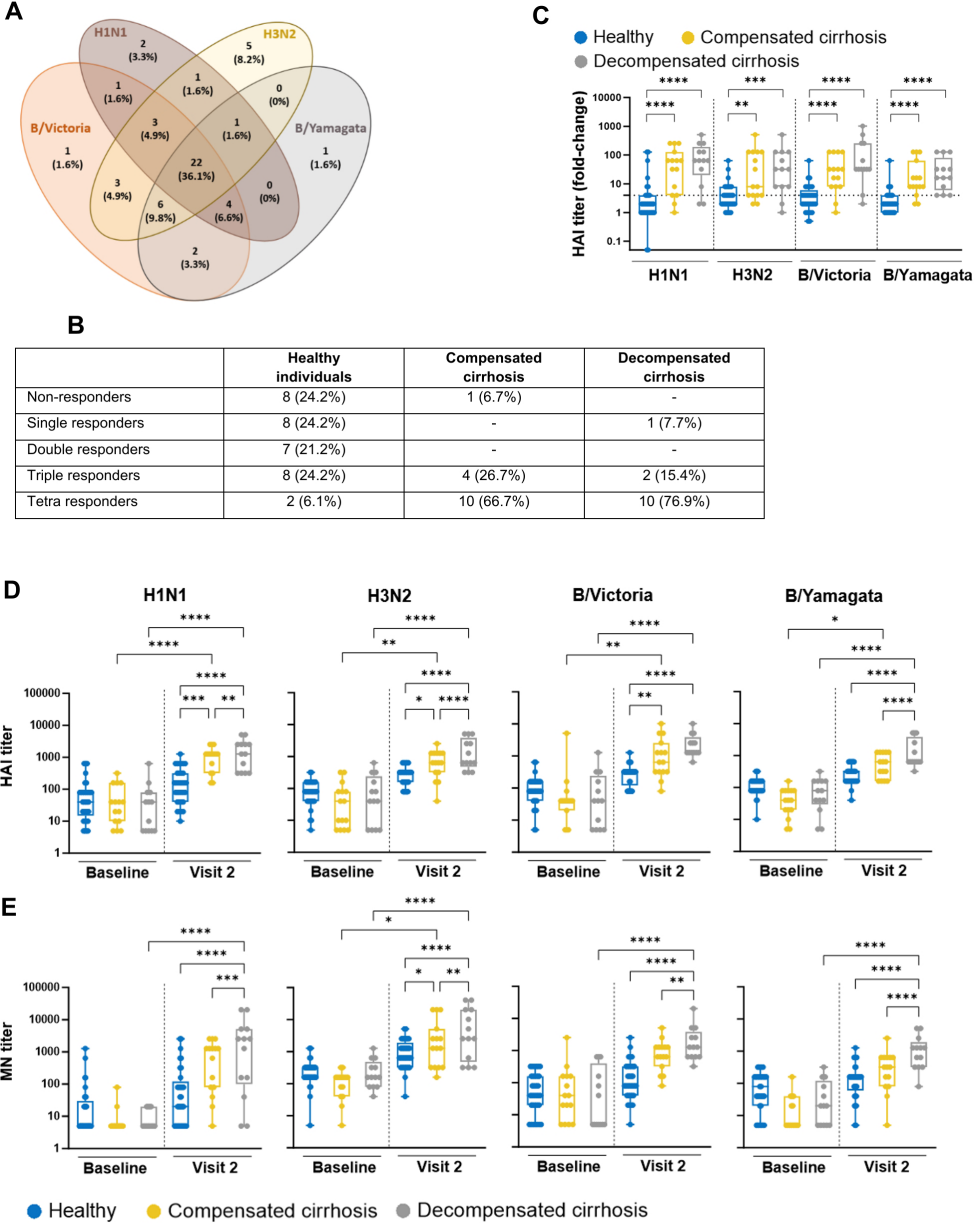
Humoral immune responses upon influenza vaccination in healthy individuals and cirrhotic patients of the 1st season. **(A)** Vaccinees of the 1st season were stratified according to their vaccine responses based on the quantification of influenza-specific antibodies in HAI assay. **(A)** Venn diagrams represent the overlapping responses against the four antigens included in the formulation. Data are presented as n (%). **(B)** Stratification of vaccinees according to their vaccine response to either none-, one-, two-, three- or four-antigens in HAI assay. **(C)** Box plots depict all individual points (minimum to maximum) with median and quartiles of HAI titers fold-change upon vaccination. Dotted line indicates the cut-off fold-change of 4. Statistical significance is based on the Mann-Whitney test. **(D)** HAI and **(E)** MN titers against each antigen of serum samples derived from vaccinees. Data are presented as box plots depicting all individual points (minimum to maximum) with median and quartiles. Two-way ANOVA with Fisher’s LSD test was applied for statistical significance (*p ≤ 0.05; **p ≤ 0.01; ***p ≤ 0.001; ****p ≤ 0.0001).

The classification of vaccine responders and non-responders is based on the fold-increase of the post-vaccination HAI antibody titer (V2) as compared to the BL titer. The serological response among all subjects was greater against the B/Victoria antigen (1^st^ season 68.8%, 2^nd^ season 58.5%), followed by H3N2 (1^st^ 67.1%, 2^nd^ 56.0%) and lastly H1N1 (1^st^: 55.7%, 2^nd^: 51.2%) as well as B/Yamagata (1^st^: 59.0%, 2^nd^: 43.9%) (**Figure 1A**, **Supplementary Figure 2A**). The identification of subjects responding to all of the antigens revealed 22 tetra responders (36.1%) for the 1^st^ season and 12 tetra responders (29.3%) for the 2^nd^ season, whereas total vaccine non-responders represented 14.8% (1^st^ season) and 22.0% (2^nd^ season), respectively (**Supplementary Figure 3**). The stratification of all subjects according to their vaccine responsiveness against either none, one, two, three, or four antigens (named non-responders, single responders, double responders, triple responders and tetra responders, respectively) is summarized in **Figure 1B** and **Supplementary Figure 2B**. Interestingly, the majority of healthy individuals were identified as non (1^st^: 24.2%, 2^nd^: 27.3%), single (1^st^: 24.8%, 2^nd^: 24.2%) or double (1^st^: 21.2%, 2^nd^: 18.2%) responders, whereas the majority of patients with liver cirrhosis were triple (1^st^: 21.4%, 2^nd^: 37.5%) or tetra (1^st^ 71.4%, 2^nd^: 62.5%) responders. Moreover, the comparison of the vaccine-induced seroconversion rates revealed differences between the study groups. Here, cirrhotic patients showed higher ratios from pre- to post-vaccine responses (*i.e*., mean fold-change) against all antigens in both seasons, as compared to healthy subjects (**Figure 1C**, **Supplementary Figure 2C**, **Supplementary Tables 8**, **9**).

To further address the significance of influenza-specific humoral immune responses in each study group, HAI geometric mean titers (GMTs) were evaluated. In the 1^st^ season, cirrhotic patients showed higher post-vaccination HAI titers for all vaccine antigens as compared to healthy subjects. Of note, those patients with decompensated cirrhosis even developed the highest titers (**Figure 1D**, **Supplementary Table 10**). Similar tendencies were observed for the 2^nd^ season, demonstrating that this pattern is independent of the influenza season and the vaccine antigenic composition (**Supplementary Figure 2D**, **Supplementary Table 11**). In addition, the comparison of the overall predicted seroprotection rate in both seasons following vaccination was comparable for all study groups (**Supplementary Tables 8**, **9**). Besides the quantification of the humoral immune responses, the functionality of neutralizing antibodies was investigated using the MN assay. For the samples taken during the 1^st^ season, post-vaccination MN titers followed the same pattern observed for significantly higher post-vaccination HAI titers in the chronic liver disease groups as compared to the healthy subjects (**Figure 1E**, **Supplementary Table 10**). The obtained observations were confirmed with the samples derived from the 2^nd^ season (**Supplementary Figure 2E**, **Supplementary Table 11**). Thus, the presented data emphasized the induction of stronger humoral responses in cirrhotic patients as compared to healthy individuals upon influenza vaccination. Of note, response was even better among those with more advanced, decompensated stage of cirrhosis. The correlation analysis of HAI and MN titers from both seasons resulted in statistically significant positive correlations for all vaccine antigens (**Supplementary Figure 4**).

### Cirrhotic patients display distinct T helper cell activation and antibody-secreting B cell responses

**Figure 2:**
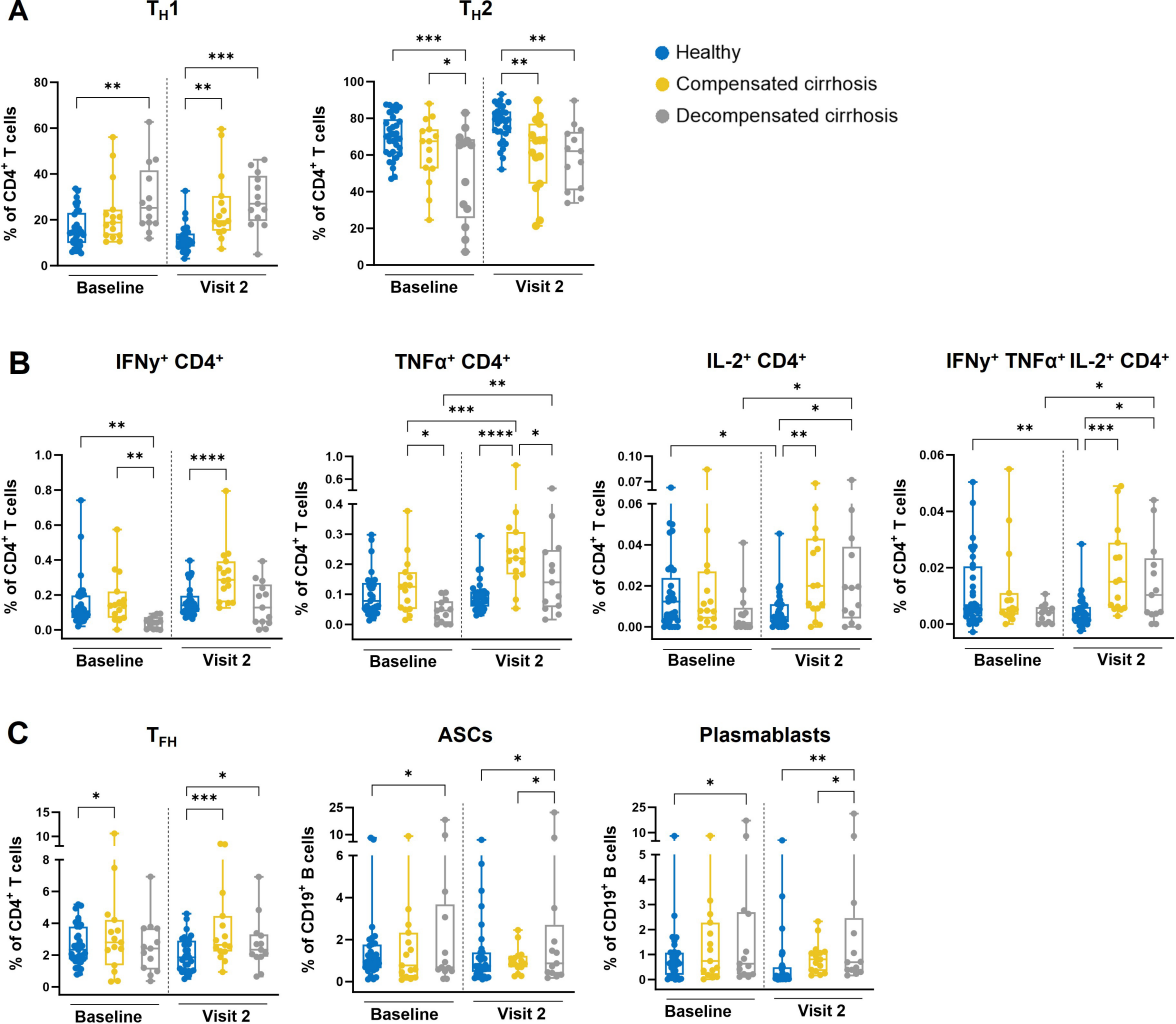
Immunophenotyping of T and B cell subsets in samples obtained during the 1st season. Cryopreserved PBMCs of vaccinees from the 1st season, before and after (V2) vaccination, were re-stimulated with the vaccine antigens. Cells were stained for surface and intracellular markers and analyzed by flow cytometry. Box plots depict all individual points (minimum to maximum) with median and quartiles for each study group. **(A)** Data are represented as frequencies of re-stimulated CD4+ T cells. TH1 cells (CD3+ CD4+ CCR6- CXCR3+). TH2 cells (CD3+ CD4+ CCR6- CXCR3-). **(B)** Data are presented as antigen-specific frequencies with subtracted background for functionality. Cytokine secreting T helper cells (CD3+ CD4+ IFNy+/IL-2+/TNFα+). **(C)** TFH cells (CD3+ CD4+ ICOS+ CXCR5+) are presented as frequencies of re-stimulated CD4+ T cells. ASCs (CD3- CD19+ CD20- CD38++ CD27+) and plasmablasts (CD3-CD19+ CD20- CD38++ CD27+ CD138-) are presented as frequencies of re-stimulated CD19+ B cells. Two-way ANOVA with Fisher’s LSD test was applied for statistical significance (*p ≤ 0.05; **p ≤ 0.01; ***p ≤ 0.001; ****p ≤ 0.0001).

The observed differences in vaccine-induced antibody responses between healthy individuals and cirrhotic patients led to the further investigation of cellular immune responses. The potential role of specific immune cell subsets was analyzed using cryopreserved PBMCs derived from BL and V2, which were re-stimulated with a mixture of the corresponding vaccine antigens. Due to the limited sample size in the 2^nd^ season, the flow cytometry analysis was confined to the 1^st^ season. The assessment of CD4^+^ T cell subsets, namely T_H_1 (known to support cell-mediated responses) and T_H_2 (known to support antibody responses), revealed different phenotypes between the study groups (**Figure 2A**). Significantly higher T_H_1 frequencies were found in the decompensated cirrhosis group at both analyzed time points, as well as after vaccination in the compensated cirrhosis group as compared to healthy individuals. In contrast, the analysis showed higher T_H_2 frequencies in the control group as compared to the chronic liver disease groups.

Since elevated frequencies of Th1 cells were especially identified among samples derived from the cirrhotic patients, the functionality of these immune cells, characterized by the production of cytokines, was subsequently explored with cell frequencies subtracted for background functionality. CD4^+^ T cells producing one cytokine (either IFNγ, TNFα, or IL-2) or three cytokines (IFNγ, TNFα and IL-2) could be identified for all groups at both time points (**Figure 2B**). Decompensated cirrhosis patients showed the lowest responses at BL, whereas after vaccination, both cirrhotic groups exhibited higher frequencies of cytokine-producing CD4^+^ T cells as compared to healthy individuals. An increased vaccine-induced expansion of cytokine-producing T cells could be predominantly found in decompensated cirrhosis patients.

Aiming a broad characterization of immune responses in cirrhotic patients, B cell responses upon antigen-re-stimulation were also analyzed by flow cytometry with a focus on the overall distribution of antibody-secreting B cells (ASCs), plasmablasts and T_FH_ cells as key B cell-supporting subsets. Increased frequencies of T_FH_ cells were detected in the cirrhotic groups, thereby mirroring the pattern observed for vaccine-induced high antibody titers. In line with this, higher frequencies of both ASCs and plasmablasts were observed in cirrhotic patients as compared to healthy individuals (**Figure 2C**).

### Cytokine patterns in healthy individuals and cirrhotic patients underpin different immune responses

**Figure 3:**
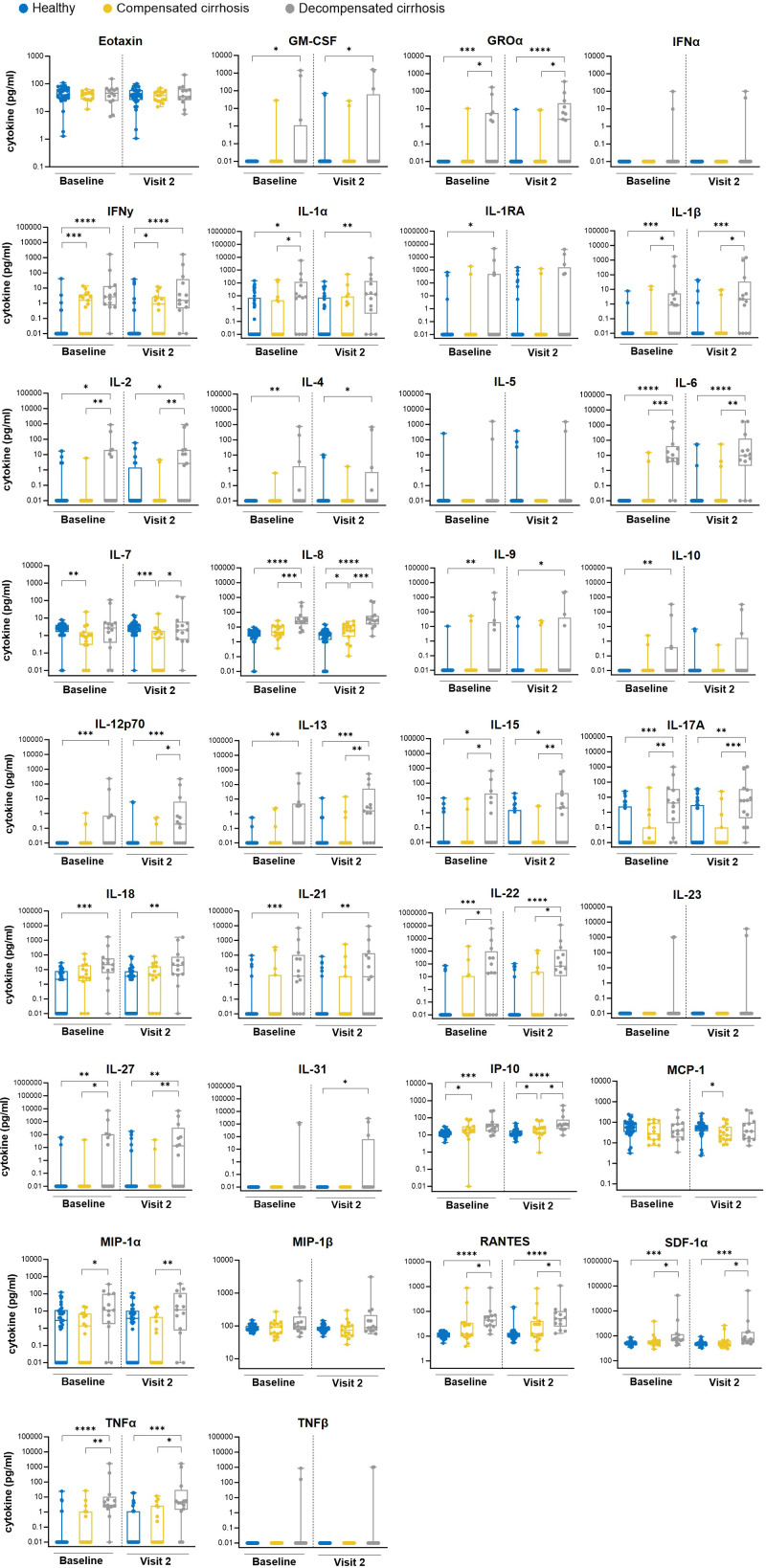
Measurement of serum cytokine levels in samples from the 1st season. Serum samples derived from vaccinees (BL and V2) of the 1st season, were tested for 34 different cytokine concentrations with the Luminex bead assay. Box plots depict all individual points (minimum to maximum) with median and quartiles. Mann-Whitney test was applied for statistical analysis (*p ≤ 0.05; **p ≤ 0.01; ***p ≤ 0.001; ****p ≤ 0.0001).

To identify soluble factors contributing to the better vaccine responses observed in cirrhotic patients, cytokine concentrations in serum samples were assessed. The analysis of samples acquired in the 1^st^ season showed distinct vaccination-independent profiles in the study groups. Comparison of cirrhotic patients with healthy subjects revealed several soluble immune factors increased especially in patients with decompensated cirrhosis, including proinflammatory chemokines and cytokines (**Figure 3**).

**Figure 4:**
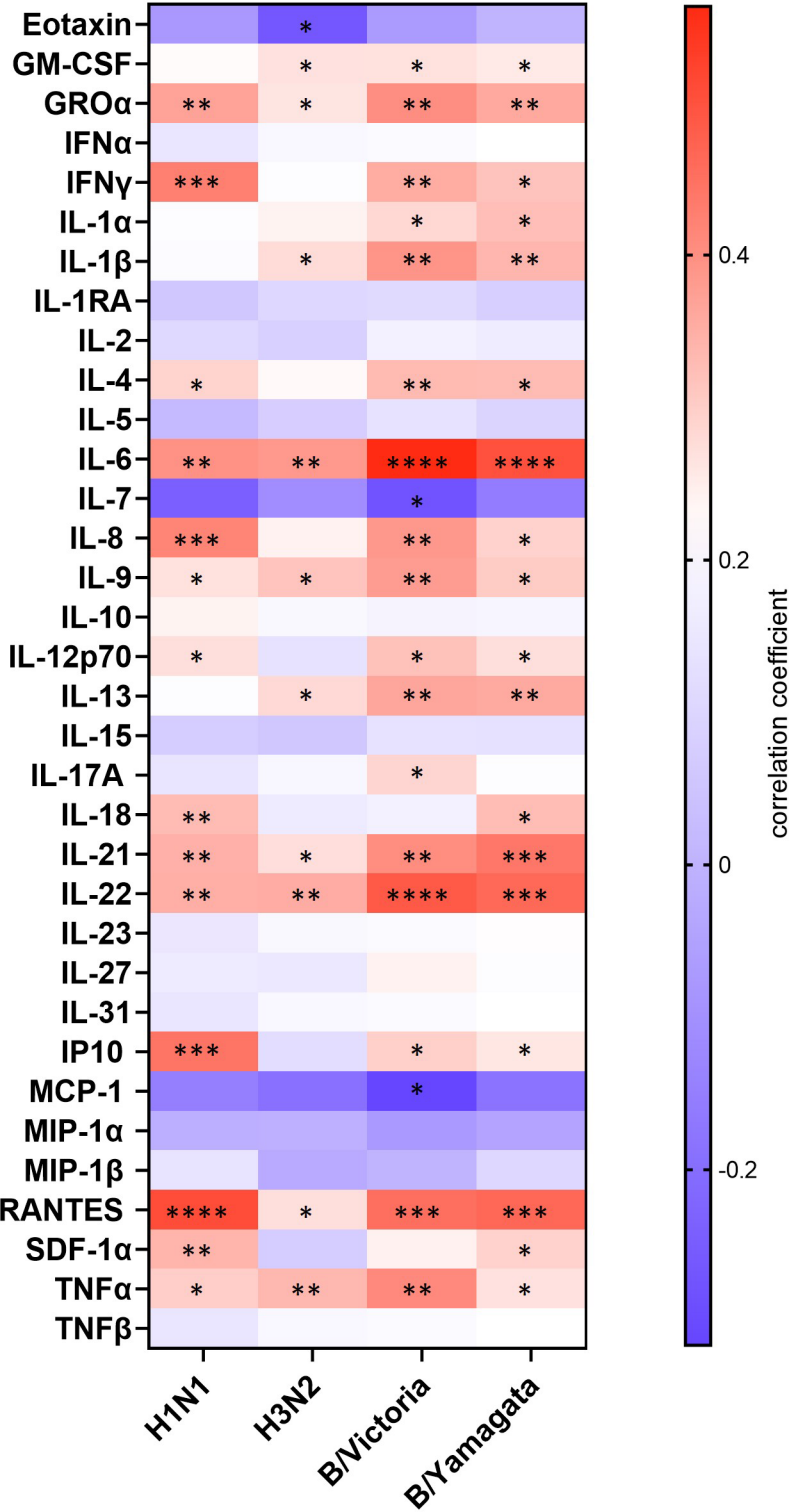
Correlation analysis of BL cytokine levels and post-vaccination HAI titers of samples obtained during the 1st season. Heat map depicts the Spearman correlation coefficient r between BL cytokine levels and post-vaccination (V2) HAI titers against each antigen separately. Data from the study groups of the 1st season were combined for analysis. Red indicates positive and blue negative associations (*p ≤ 0.05; **p ≤ 0.01; ***p ≤ 0.001; ****p ≤ 0.0001).

As observed above, datasets on HAI and MN titers showed enhanced responses in cirrhotic patients compared to healthy individuals. Thus, to identify putative BL factors influencing responses to vaccination a correlation analysis of BL cytokine levels and post-vaccination HAI titers was performed, using combined data derived from all participants of the 1^st^ season. Several cytokines significantly correlate with vaccine-induced antibody titers (**Figure 4**). Negative correlations were observed for Eotaxin, IL-7 and MCP-1 against at least one antigen, whereas strong positive correlations were found for IFNγ, TNFα, IL-6, IL-21 and IL-22. These data suggest that enhanced antibody responses in cirrhotic patients might be shaped by a preexistent inflammatory environment that subsequently leads to a strong immune activation upon vaccine administration.

### Circulating metabolites are associated with post-vaccination humoral immune responses

**Figure 5:**
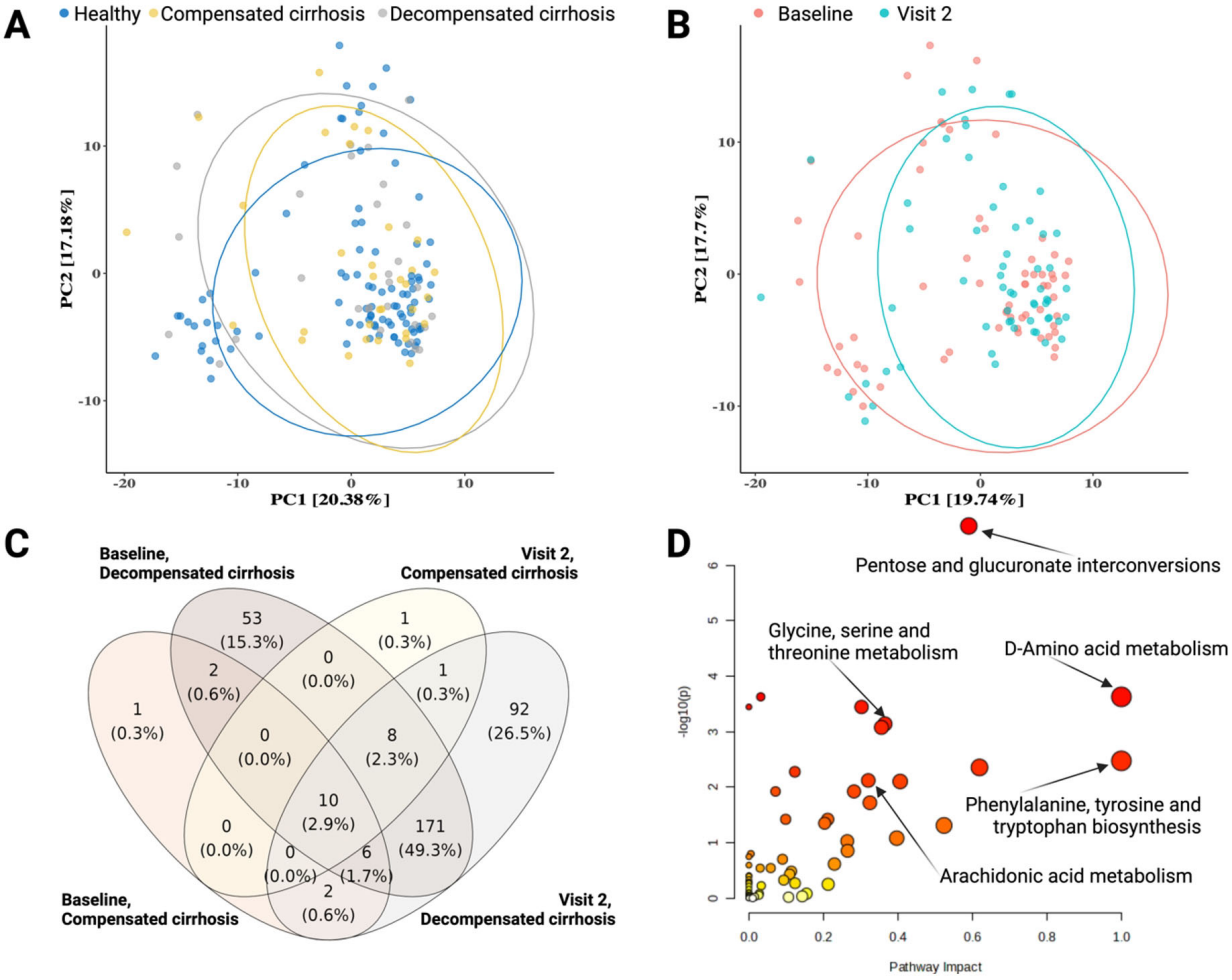
Circulating metabolite profiles of vaccinees in samples obtained during the 1st season. **(A, B)** The PCA plots represent the metabolite profiles between cirrhotic patients and healthy subjects, and between pre- and post-vaccination. **(C)** The number of metabolites that were significantly associated with cirrhotic conditions compared to healthy subjects at BL and V2 after adjusting for age and sex factors. P-adj <0.05. **(D)** Pathway analysis of metabolites that were significantly associated with cirrhotic conditions at either BL or V2.

To further explore the underlying mechanisms leading to different immune responses, we analyzed 786 circulating metabolites in serum samples at BL and V2. While individuals’ metabolite profiles were grouped based on their health conditions (**Figure 5A**), there was no clear differentiation in metabolite profiles between pre- and post-vaccination samples (**Figure 5B**). This confirms the previous observation in the cell subset and cytokine profiles (**Figures 2**, **3**), in which the molecular signatures in cirrhotic patients distinctly differ from those in healthy individuals. Approximately half of the measured metabolites (347 metabolites, 44.2%) were significantly associated with cirrhotic conditions compared to healthy individuals at BL and post-vaccination (padj <0.05) in the 1^st^ season (**Figure 5C**). A similar observation was affirmed in the metabolite analysis in the 2^nd^ season (**Supplementary Figures 5A–C**). Those metabolites, which were significantly associated to cirrhotic conditions in the 1^st^ season, are involved in different pathways such as pentose and glucuronate interconversions, glycine, serine and threonine metabolism, or arachidonic acid metabolism (**Figure 5D****).**

**Figure 6:**
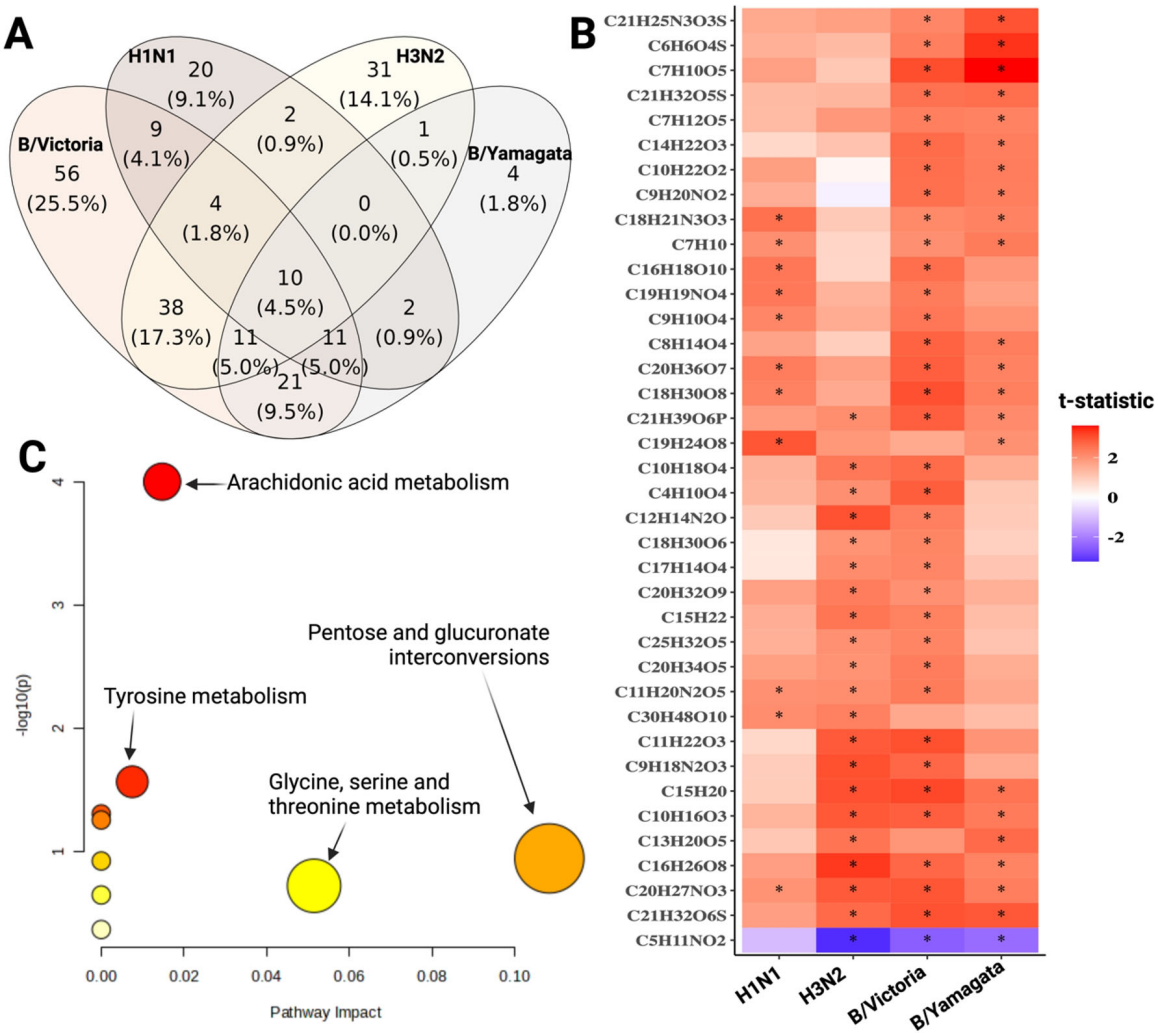
Association analysis of BL metabolite levels and post-vaccination HAI titers in samples of the 1st season (2019-2020). **(A)** The number of metabolites at BL that were significantly associated with post-vaccination (V2) HAI titer against each antigen separately after adjusting for age, sex, and disease conditions. P-value <0.05. **(B)** The heat map depicts the association between BL metabolite levels and post-vaccination (visit 2) HAI titers against each antigen separately. The association was calculated using the t-statistic of the linear association model between BL metabolite levels and post-vaccination (V2) HAI titers after adjusting for the influence of sex, age, and disease conditions among participants. The shown metabolites are significantly associated with HAI titer in at least 2 antigens in the 1st season and showed a consistent trend in at least 75% across antigens and both seasons. The asterisk indicates a significant association (p-value <0.05). Red indicates positive and blue negative associations. **(C)** Pathway analysis of 38 metabolites that were associated with post-vaccination HAI titers shown in **(B)**.

In total, 220 and 147 metabolites were significantly associated with the post-vaccination (V2) HAI titer of at least one antigen in the 1^st^ and 2^nd^ season, respectively (**Figure 6A**, **Supplementary Figures 5D**, p-value<0.05). Due to the limited sample size in the 2^nd^ season and the heterogeneity in metabolite profiles, we focused on the 38 metabolites (17.3%) out of the 220 metabolites in the 1^st^ season that showed consistent associations with post-vaccination HAI titers across different antigens in both seasons (**Figure 6B**). Those metabolites also participate in the pentose and glucuronate interconversions, glycine, serine and threonine metabolism, and arachidonic acid metabolism pathways (**Figure 6C**, **Supplementary Table 12****).** In particular, C20H34O5 can participate in the arachidonic acid metabolism pathway as prostaglandin E1, prostaglandin H1, or other trienoic fatty acids, since they have the same chemical formula but different molecular structures (14). Similarly, C5H11NO2 could be valine or betaine which is an intermediate metabolite involved in the glycine, serine, and threonine metabolism. While the BL level of C20H34O5 was positively associated with the post-vaccination HAI titers across all influenza antigens, the level of C20H34O5 at BL was significantly upregulated in the cirrhotic patients as compared to the healthy subjects (**Supplementary Figure 6**).

### Impact of clinical parameters on vaccine-induced immune responses

To further investigate if the observed differences in vaccine-induced immune responses could be linked to clinical and routine laboratory BL parameters, clinical data from the 1^st^ season were analyzed. Several BL parameters significantly differed between the high and low/non vaccine responders (**Supplementary Table 13**). Indicators of liver functions (model of end stage liver disease [MELD-Score] (15) and cholinesterase level [kU/L, CHE]) and antibody-related routine parameters (IgG level and lymphocyte count) were correlated with post-vaccination (V2) HAI titers of cirrhotic patients. In these analyses, IgG serum level did not significantly correlate with HAI titers. The lymphocyte counts were negatively correlated with HAI titers against antigens of H1N1 and B/Victoria. Regarding indicators of liver functions, a low CHE was significantly associated with higher HAI titers against three out of four antigens and higher MELD scores significantly correlated with higher HAI titers against antigens of B/Victoria and B/Yamagata (**Supplementary Figure 7**). Thus, the obtained results underpin the stronger vaccine response in patients with advanced liver cirrhosis.

## Discussion

Viral infections caused by non-hepatotropic viruses like influenza virus represent a particular threat in patients with liver cirrhosis and are associated with increased hospitalization risk and high mortality rates (4). Beside severe respiratory symptoms, influenza virus infections are also associated with the development of hepatic decompensation and ACLF.

Despite clear recommendations, vaccination coverage against influenza among liver cirrhosis patients remains suboptimal (8). Although few reports about the effectiveness of influenza vaccination in cirrhotic patients exist, this is the first study that addresses an in-depth analysis of both humoral and cell-mediated immunity, in combination with BL signatures to tetravalent inactivated influenza vaccines among compensated and decompensated cirrhosis patients. In support of the development of vaccine-induced antibodies upon influenza vaccination in cirrhotic patients provided by previous studies (16, 17), our data show that the tetravalent influenza vaccine triggers not only higher seroconversion rates, but also higher post-vaccination HAI GMTs in cirrhotic patients as compared to healthy individuals. Importantly, vaccine response showed a positive correlation with stage of liver disease and, thus, was even better among those decompensated cirrhosis that are the most vulnerable group for infections.

The analysis of the cellular and cytokine immune profiles in cirrhotic patients revealed evidence for a linkage between the immune alterations present in cirrhosis (CAID) and the observed enhanced humoral response in these patients. The severity of CAID is precipitated by a continuous spectrum of immune alterations that increase with higher stages of liver cirrhosis. These immunological alterations are caused by two main mechanisms. First, injured, dying or stressed hepatocytes release damage-associated molecular patterns (DAMP’s) and second, portal hypertension in patients with liver cirrhosis leads to an increased translocation of pathogen-associated molecular patterns (PAMPs) from the gut to the blood stream (2). In patients with cirrhosis, but without extrahepatic organ failure, an increased expression of surface antigens indicating the activation of circulating immune cells and an increment of proinflammatory cytokines occur (1). This preexisting hyperinflammatory immune profile, particularly in decompensated liver cirrhosis, might promote the high antibody titers observed following vaccination. The inflammatory status in cirrhotic patients was confirmed by the detection of soluble serum factors. Strong positive correlations between the pre-vaccination proinflammatory cytokines and post-vaccination HAI titers were observed. For some of the soluble immune factors, including IFNγ, TNFα, IL-21 and IL-6, positive associations with influenza-specific antibody responses following vaccination or infection were reported (18–21). The importance of robust T cell responses and their secreted cytokines to orchestrate a balance between pro- and anti-inflammatory processes during the encounter with influenza viruses is highlighted by the positive correlation of cell-mediated immunity with improved disease protection (22, 23). In addition to a hyperinflammatory baseline environment, several non–mutually exclusive mechanisms may contribute to the unexpectedly strong humoral responses in cirrhosis. These include altered vaccine pharmacokinetics (e.g., changes in distribution/clearance that may affect antigen exposure kinetics), differences in antigen uptake/processing and presentation, and/or impaired regulatory immune pathways that could lower suppression of vaccine-driven responses (24–26). Future studies integrating pharmacokinetic and functional antigen-presentation assays, as well as regulatory cell profiling, will be required to delineate the relative contribution of these mechanisms.

In our study, the frequencies of CD4^+^ Th1 cells were significantly enhanced in cirrhotic groups as compared to the healthy individuals. Previous studies have reported that in the course of cirrhosis or viral infections, the T_H_1 population and its cytokines promote inflammation (27, 28). Furthermore, previous influenza studies showed that T_H_1 cells, secreting TNFα, IFNγ and/or IL-2 upon activation, are essential for the development of the T cell memory compartment for long-term protection against influenza virus infections (29, 30). Therefore, the obtained data suggest that a more efficient activation of antigen-specific effector T helper cells in cirrhotic patients might have led to stronger vaccine-induced humoral immune responses. However, a significant vaccine-induced increase of the respective serum cytokine levels could not be detected for the study groups. Although no differences were observed, there might be a difference in the early inductive phase upon vaccination. It has been demonstrated that the peak of cytokine production is within the first hours and days of post-vaccination (31). Since our study only assessed cellular responses approximately one month after vaccination, earlier time points might give further hints towards a putative differential initiation of vaccine responses. Correlation of protective influenza-specific immunity was also shown for B cell responses, including T_FH_ cells and plasmablasts (32–34). In our study, higher frequencies of both cell subsets were observed in cirrhotic patients as compared to healthy subjects upon vaccination. This might also be promoted by the aforementioned enhanced proinflammatory status in the patients.

Metabolite profile analysis further contributed to understanding the immune responses to influenza vaccines, as well as the difference between cirrhotic and healthy individuals. Cirrhotic patients had distinct circulated metabolite profiles compared to healthy individuals. This observation aligns with previous studies on metabolite signatures associated with cirrhosis and its complications (35, 36). For instance, a high level of arachidonic acid is an early indicator of inflammation and irreversible in non-alcoholic fatty liver disease progress (37). Liver cirrhosis patients have increased BL inflammatory profiles (38) and abnormal arachidonic acid metabolism (39). In this study, metabolites involved in the polyunsaturated fatty acid arachidonic acid metabolism, such as prostaglandins or trienoic fatty acids (C20H34O5), were upregulated in cirrhotic patients and were also positively associated with the post-vaccination HAI titer across the different antigens. Intriguingly, a high arachidonic acid diet in healthy adults could elevate the post-immunization cell proliferation in response to an influenza vaccine compared to the basal diet (40). Metabolites derived from arachidonic acid metabolism not only contribute to inflammation but also resolve inflammation and promote and modulate type 2 immune responses (41). Arachidonic acid also mediates the protection induced by the *Schistosoma mansoni* vaccine in mice (42). Thus, the upregulation of arachidonic acid metabolism due to cirrhotic stages the cirrhotic patients might contribute to a better vaccine-induced responsiveness in cirrhotic patients as compared to healthy individuals. By contrast, the levels of other metabolites, such as valine or betaine (C5H11NO2) at BL were negatively associated with post-vaccination HAI titers. While amino acid profiles are abnormal in patients with advanced cirrhosis (43), an increased level of branched amino acids, e.g. valine and betaine, could negatively impact lung immunity against the influenza virus and enhance the severity of influenza virus infection (44). Since polyunsaturated fatty acid signatures were associated with a robust influenza vaccine in young healthy individuals (45), further studies are necessary to examine how the intake of arachidonic acid as immunomodulatory molecules can increase response to influenza vaccines.

Our study has some limitations. Next to the above-mentioned differences in the study cohorts (*e.g*., range of age between cirrhotic patients vs. healthy subjects without liver disease), further factors that were not investigated or assessed in this study can influence the vaccine response. For example, genetic factors (single nucleotide polymorphisms in HLA, cytokines, or other immune-related molecules), differences in the microbiome, or the hormonal milieu are known to influence immune responses upon influenza vaccination (46). Moreover, the higher coverage of previous influenza vaccinations in the healthy group as compared to the cirrhotic patients might further contribute to the observed differences in the vaccine-induced immune responses, since it was reported that pre-existing antibodies can attenuate immune responses of consecutive immunizations (47, 48). In line with this directionality, an exploratory responder-phenotype comparison in this study, restricted to healthy controls, showed that non-responsiveness was more frequent among individuals reporting previous influenza vaccination. Subgroup analyses involving decompensated cirrhosis (n=3) of the second season should be interpreted cautiously due to limited sample size and are therefore considered exploratory and should be confirmed in larger cohorts. In addition, cellular immune profiling was performed approximately one month after vaccination. As early cytokine and T cell activation events are most prominent within the first days after immunization, earlier sampling might have provided additional insight into the induction phase of the response. However, the WHO recommendation for the influenza vaccine differed between 2019/2020 and 2020/2021 and the trend emerging from the results of the 1^st^ season can be validated with the 2^nd^ season, demonstrating that they are independent of the vaccine antigenic composition.

In conclusion, our study shows that influenza vaccination is effective in both compensated and, more importantly, decompensated liver cirrhosis patients, thereby underpinning the potential clinical benefit by reducing the risk of severe influenza virus infections in this high-risk group.

## Data Availability

The original contributions presented in the study are publicly available. This data can be found here: https://doi.org/10.5281/zenodo.19480454.

## Glossary

ACLF: acute-on-chronic liver failure
ALT: alanine aminotransferase
ASC: antibody-secreting B cell
AST: aspartate aminotransferase
BL: baseline
body mass index: BMI
CAID: cirrhosis-associated immune dysfunction
CHE: cholinesterase
CLIF-C: CLIF consortium organ failure score
CO_2_: carbon monoxide
GM-CSF: granulocyte macrophage colony-stimulating factor
GMT: geometric mean titer
GROα: growth-regulated protein alpha
HAI: hemagglutination inhibition
HLA: human leukocyte antigens
HMDB: Human Metabolome Database
IFNα: interferon alpha
IFNγ: interferon gamma
IL-1α: interleukin 1-alpha
IL-1β: interleukin 1-beta
IL-1RA: interleukin 1 receptor antagonist
IL-2: interleukin 2
IL-4: interleukin 4
IL-5: interleukin 5
IL-6: interleukin 6
IL-7: interleukin 7
IL-8: interleukin 8
IL-9: interleukin 9
IL-10: interleukin 10
IL-13: interleukin 13
IL-15: interleukin 15
IL-17A: interleukin 17A
IL-18: interleukin 18
IL-21: interleukin 21
IL-22: interleukin 22
IL-23: interleukin 23
IL-27: interleukin 27
IL-31: interleukin 31
IP10: interferon-gamma induced protein 10
INR: international normalized ratio
KEGG: Kyoto Encyclopedia of Genes and Genomes
MCP-1: Monocyte Chemoattractant Protein-1
MELD: model of end stage liver disease
MIP-1α: Macrophage Inflammatory Protein-1 Alpha
MIP-1β: Macrophage Inflammatory Protein-1 Beta
MN: microneutralization
PBMC: peripheral blood mononuclear cell
RANTES: Regulated And Normal T cell Expressed an Secreted
SDF-1α: Stromal cell-derived factor 1 alpha
T_FH_ cell: T Follicular Helper Cell
T_H_1 cell: T helper 1 cell
T_H_2 cell: T helper 2 cell
TNFα: tumor necrosis factor-α
TNFβ: tumor necrosis factor-beta
V1: visit 1
V2: visit 2
V3: visit 3
WHO: World Health Organization.
