# Supplementary material for: Particularly strong immune response to influenza vaccination in patients with decompensated liver cirrhosis linked to systemic inflammation
Source: Front Immunol. 2026 Apr 22;17:1734093. doi: 10.3389/fimmu.2026.1734093 (PMC13143674; doi:10.3389/fimmu.2026.1734093)
Supplement: Supplementary file 1 [file DataSheet1.docx]

**Details of laboratory testing’s**

**Preparation of antigens and infectious virus stocks**

The following antigens were used for the hemagglutination inhibition (HAI) assay and the antigen re-stimulation of PBMCs for flow cytometric analysis: A/Brisbane/02/2018 H1N1 IVR-190 (season 2019/20), A/Kansas/14/2017 H3N2 NYMC X-327 (season 2019/20), B/Maryland/15/2016 Victoria lineage NYMC BX-69A (season 2019/20), B/Phuket/3073/2013 Yamagata lineage (both seasons), A/Guangdong-Maonan/SWL1536/2019 H1N1 CNIC-1909 (season 2020/21), A/Hong Kong/2671/2019 H3N2 NIB-121 (season 2020/21) and B/Washington/02/2019 Victoria lineage (season 2020/21). Infectious virus preparations for microneutralization (MN) assay were propagated in the chorioallantoic cavity of 10-day-old embryonated eggs for 48 h at 37°C, as described previously ^1^.

**Classification of vaccine responders and non-responders with the HAI assay**

HAI assay was performed as described previously ^2^. Serum samples were treated with receptor-destroying enzyme (RDE) at a 1:4 ratio (1 volume serum + 4 volumes RDE) overnight at 37°C, heat-inactivated for 30 min at 56°C, and diluted 1:1 with 0.9% NaCl prior to testing. Turkey red blood cells (RBCs) were washed and prepared as a 1% suspension in 0.9% NaCl. Viral antigens were standardized to 4 hemagglutination units (HAU) per 25 µl by back titration. RDE-treated sera were serially twofold diluted in 1x PBS in V-bottom 96-well plates (starting dilution 1:10; 25 µl/well) and mixed 1:1 with standardized antigen (25 µl/well). After 15 min incubation at room temperature, 50 µl of 1% RBC suspension was added and plates were incubated until the RBC control formed a tight button. Assay conditions and controls included RBC-only (cell control) wells, antigen back-titration to confirm 4 HAU per well, a negative serum control, and where available a reference/positive control serum, run in parallel on each plate to ensure assay validity.

The HAI titer was defined as the reciprocal value of the highest serum dilution at which complete inhibition of hemagglutination was achieved. HAI antibody titers of ≥40 were considered seroprotective. According to the Committee for Proprietary Medicinal Products criteria, a positive seroresponse to vaccination has to fulfill a ≥4-fold increased post-vaccination titer with a pre-vaccination titer ≥10 or a post-vaccination titer of ≥40 with a pre-vaccination titer <10 ^3^. HAI titers below the limit of detection (<10) were assigned as 5 for subsequent calculations.

In this study, “particularly strong” immune responses refer to participants who fulfilled the predefined responder criteria across multiple vaccine components. Specifically, individuals classified as triple- or tetra-responders achieved seroconversion (defined as a ≥4-fold increase in HAI titer from baseline) against three or all four influenza vaccine strains included in the seasonal formulation, respectively.

**Measurement of MN antibody titers**

The MN assay was performed as described previously ^4^. For neutralization, RDE-treated sera were serially twofold diluted in virus diluent starting at 1:20 and mixed with 100×TCID50 virus. After 1 h incubation at 37°C, serum–virus mixtures were added to confluent MDCK monolayers (1.5×10⁴ cells/well) in flat-bottom 96-well plates and incubated for 18–20 h at 37°C, 5% CO₂. Infected cells were fixed with cold 80% acetone and viral nucleoprotein was detected by ELISA using mouse anti-influenza A nucleoprotein (Bio-Rad; 1:1000) or mouse anti-influenza B nucleoprotein (Bio-Rad; 1:500) followed by HRP-conjugated secondary antibody and OPD development. Assay conditions and controls included cell-only wells (background control), virus-only wells (infection control), and virus back-titration to verify the infectious input. MN titers were defined by calculating the cut-off value to determine a 50% neutralization of the virus. The first reciprocal serum dilution corresponding to 50% neutralization represents the antibody titer. MN titers below the limit of detection (<20) were assigned as 5 for subsequent calculations.

**Flow cytometry analysis**

For immunophenotyping of vaccinees, cryopreserved PBMCs were thawed and rested for 2 h at 37°C with 5% CO_2_. Subsequently, cells were co-stimulated with purified anti-human CD28 (5 µg/ml) and CD49d (5 µg/ml) and re-stimulated with a mixture consisting of all four individual vaccine antigens (2.5 µg/ml per each antigen). The cells were incubated for 4 h, followed by the addition of monensin (6 µg/ml) and brefeldin A (5 µg/ml) as cytokine secretion inhibitors. After 12 h of incubation, the cells were stained in volumes of 50 µl at 4°C for 25 min, protected from light, using the antibodies listed in **Supplementary Table 1**. Prior to intracellular staining, cells were permeabilized using the Cytofix/Cytoperm kit (BD) or the FOXP3 staining kit (eBioscience) according to the manufacturer’s protocol. Stained cells were acquired with a BD FACSymphony™ A5 Cell Analyzer and evaluated with the software FlowJo (BD, version 10.8.1). Assay conditions and controls included analysis of unstimulated (‘ex vivo’) PBMCs in parallel with antigen re-stimulated PBMCs (stimulation with a mixture of the four vaccine antigens), with unstimulated samples serving as the background/negative control for activation and cytokine readouts.Fluorescence spillover was corrected using single-stained compensation controls (BD CompBeads; ArC™ amine-reactive compensation beads). Data quality control and gating included exclusion of acquisition instability (time gate), doublets (singlet gating), and dead cells prior to downstream subset analysis. The gating strategy is shown in **Supplementary Figure 1.**

**Serum cytokine immunoprofiling**

The measurement of cytokine and chemokine concentrations in serum samples was performed using the Cytokine & Chemokine 34-Plex Human ProcartaPlex™ Panel 1A (Thermo Fisher), following the manufacturer’s protocol, including the kit-provided standards and assay control wells required for concentration determination and quality control. The calculation of the cytokine concentrations was done with the Invitrogen ProcartaPlex Analysis online application. Concentrations below the detection limit were assigned as 0.01 pg/ml for subsequent calculations.

**Circulating metabolite profiling**

The circulating metabolite concentrations in serum samples were measured by using the untargeted metabolic platform with mass spectrometry technique (General Metabolics). Raw metabolite profile data were centroided, merged, and recalibrated using the MATLAB software ^5^. Metabolites were identified based on the mass-to-charge ratio (ion m/z) and isotopic correlation patterns using the Human Metabolome Database (HMDB) ^6^. The duplicate peak intensities were averaged and normalized using moving median normalization. A total of 1600 metabolites were measured and annotated. According to the HMDB, 786 out of these 1600 metabolites are endogenous metabolites and were used for further analysis. These metabolites were then annotated against multiple databases (HMDB, KEGG, and Chemical Entities of Biological Interest (ChEBI)) and retained all plausible matches.

In the downstream metabolite analysis, since a “particularly strong” immune responder was defined as an individual mounting strong responses to three or four viral antigens, we used the ≥75% directional consistency threshold (i.e., concordance in at least 3 of 4 antigens) to prioritise metabolites whose associations with HAI titers were robust across both antigens and seasons.

**References**

1. Brauer R, Chen P. Influenza virus propagation in embryonated chicken eggs. J Vis Exp 2015.

2. Zacour M, Ward BJ, Brewer A, et al. Standardization of Hemagglutination Inhibition Assay for Influenza Serology Allows for High Reproducibility between Laboratories. Clinical and Vaccine Immunology 2016;23:236-242.

3. Trombetta CM, Perini D, Mather S, et al. Overview of Serological Techniques for Influenza Vaccine Evaluation: Past, Present and Future. Vaccines (Basel) 2014;2:707-34.

4. World Health Organization (WHO), <https://www.who.int/publications/i/item/serological-diagnosis-of-influenza-by-microneutralization-assay> (Accessed 02 July 2023).

5. Fuhrer T, Heer D, Begemann B, et al. High-throughput, accurate mass metabolome profiling of cellular extracts by flow injection-time-of-flight mass spectrometry. Anal Chem 2011;83:7074-80.

6. Wishart DS, Feunang YD, Marcu A, et al. HMDB 4.0: the human metabolome database for 2018. Nucleic Acids Res 2018;46:D608-d617.

7. Jalan R, Pavesi M, Saliba F, et al. The CLIF Consortium Acute Decompensation score (CLIF-C ADs) for prognosis of hospitalised cirrhotic patients without acute-on-chronic liver failure. J Hepatol 2015;62:831-40.

**Supplementary Figure 1**

**A**

**
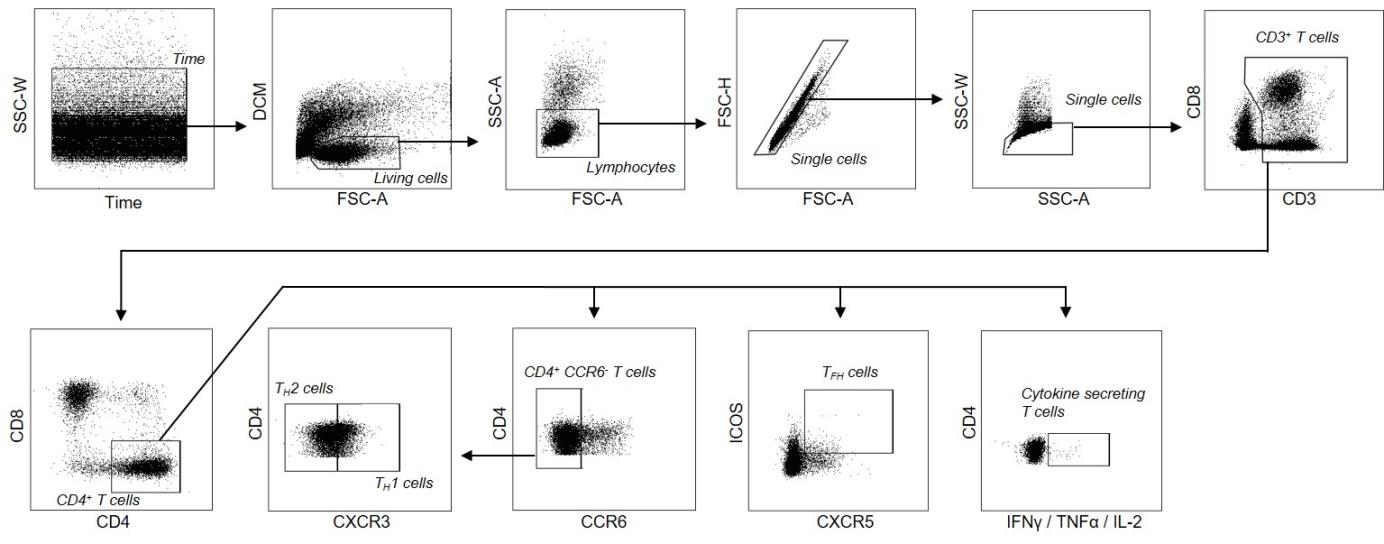
**

**B**

**
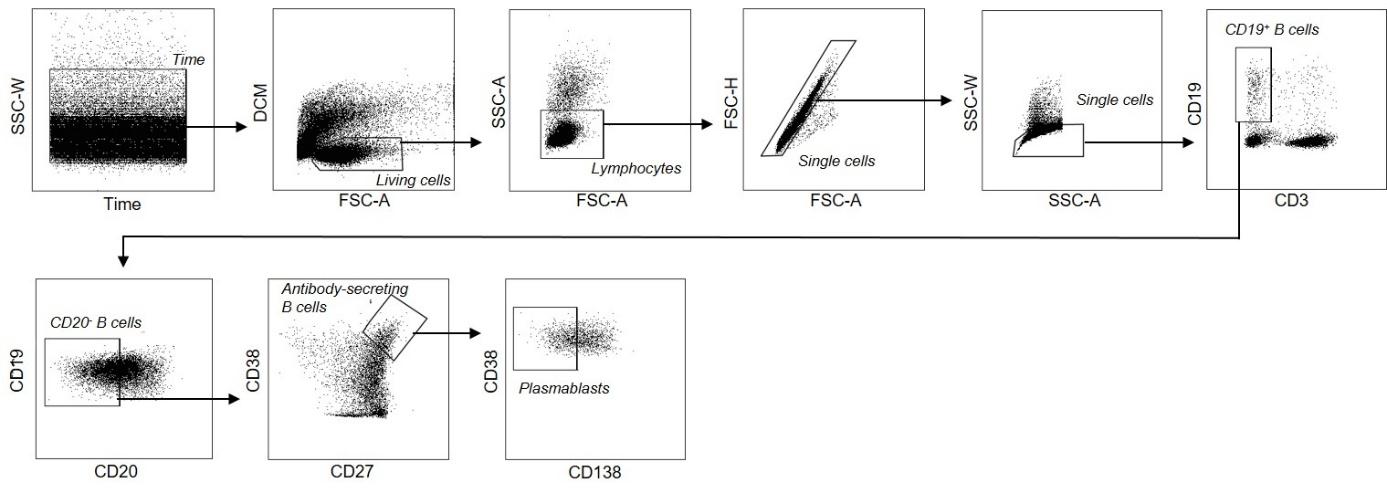
**

**Supplementary Figure 1: Gating strategy for flow cytometry analysis.** (**A**) T cell Panel. (**B**) B cell Panel.

**Supplementary Figure 2**


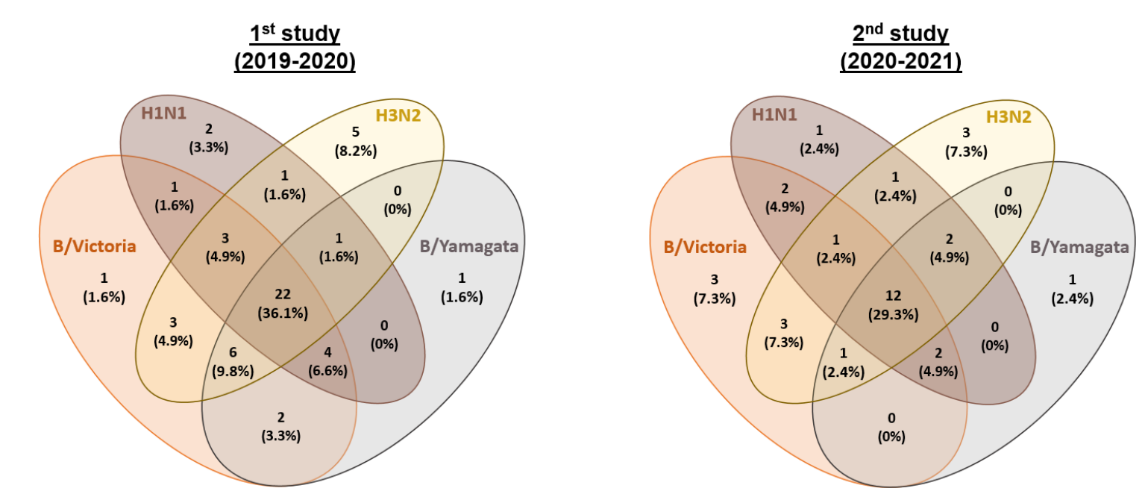

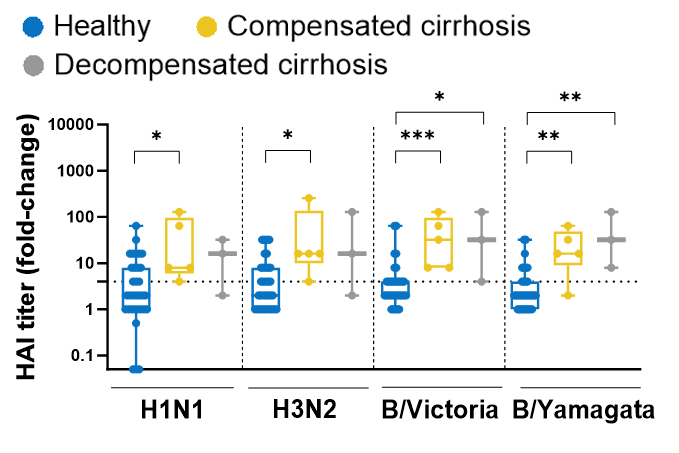


**C**

**A**

**B**

|  | **Healthy**  **subjects** | **Compensated**  **cirrhosis** | **Decompensated cirrhosis** |
| --- | --- | --- | --- |
| Non-responders | 9 (27.3%) | - | - |
| Single responders | 8 (24.2%) | - | - |
| Double responders | 6 (18.2%) | - | - |
| Triple responders | 3 (9.1%) | 1 (20%) | 2 (67%) |
| Tetra responders | 7 (21.2%) | 4 (80%) | 1 (33%) |

**D**


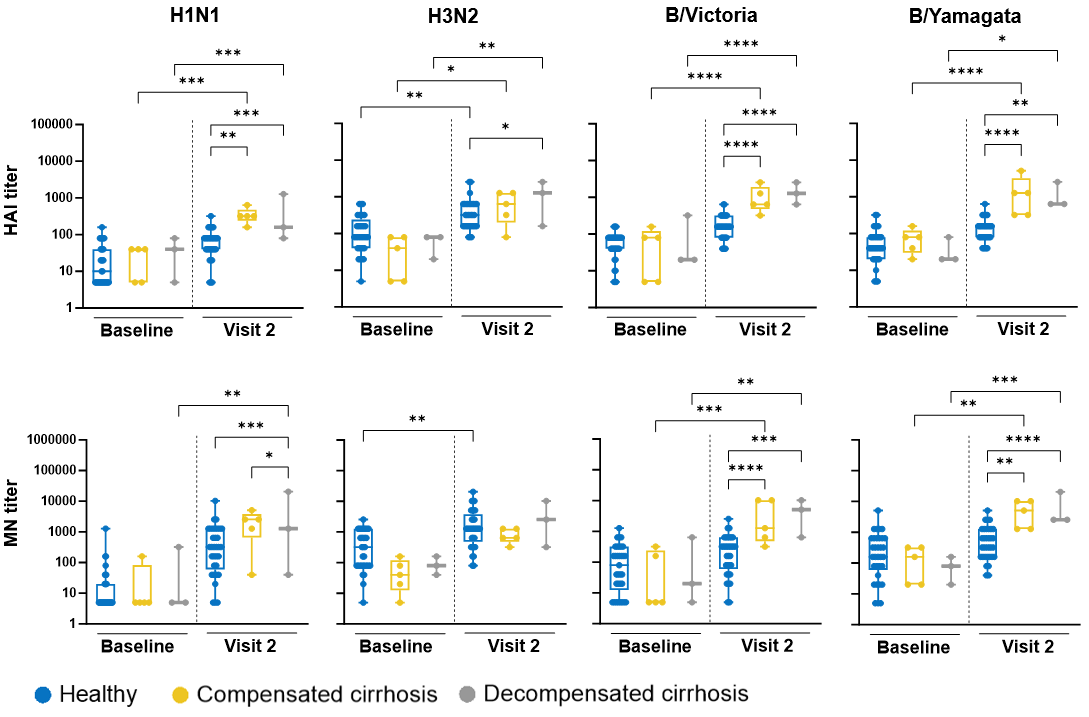


**E**

**Supplementary Figure 2: Humoral immune responses upon influenza vaccination in healthy subjects and cirrhotic patients of the 2^nd^ season. (A)** Vaccinees of the 2^nd^ season were stratified according to their vaccine responses based on the quantification of influenza-specific antibodies in HAI assay. Venn diagrams represent the overlapping responses against the four antigens included in the formulation. Data are presented as n (%). **(B)** Stratification of vaccinees according to their vaccine response to either none-, one-, two-, three- or four antigens in HAI assay. **(C)** Box plots depict all individual points (min to max) with median and quartiles of HAI titers fold-change upon vaccination. Dotted line indicates the cut-off fold-change of 4. Statistical significance is based on the Mann-Whitney test. **(D)** HAI and **(E)** MN titers against each antigen of serum samples derived from vaccinees. Data are presented as box plots depicting all individual points (minimum to maximum) with median and quartiles. Two-way ANOVA with Fisher’s LSD test was applied for statistical significance (* p ≤ 0.05; ** p ≤ 0.01; *** p ≤ 0.001; **** p ≤ 0.0001).

**Supplementary Figure 3**


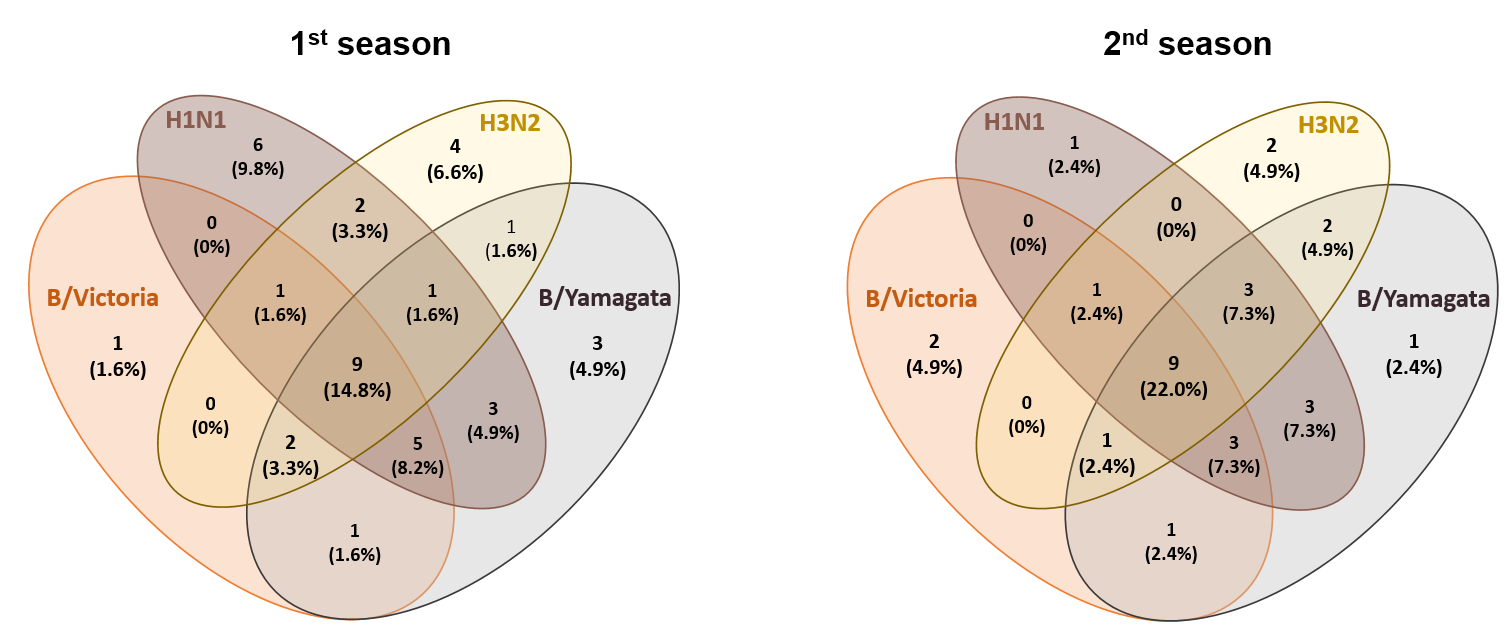


**Supplementary Figure 3: Stratification of vaccine non-responders.** Vaccinees of the 1^st^ and 2^nd^ season were stratified according to their vaccine non-responsiveness based on the quantification of influenza-specific antibodies in HAI assay. Non-responders are not fulfilling a ≥ 4-fold increased post-vaccination (visit 2) titer with a pre-vaccination titer ≥ 10 or a post-vaccination (visit 2) titer of ≥ 40 with a pre-vaccination titer <10. Data are presented as n (%). Venn diagrams represent the overlapping non-responses against the four antigens.

**Supplementary Figure 4**


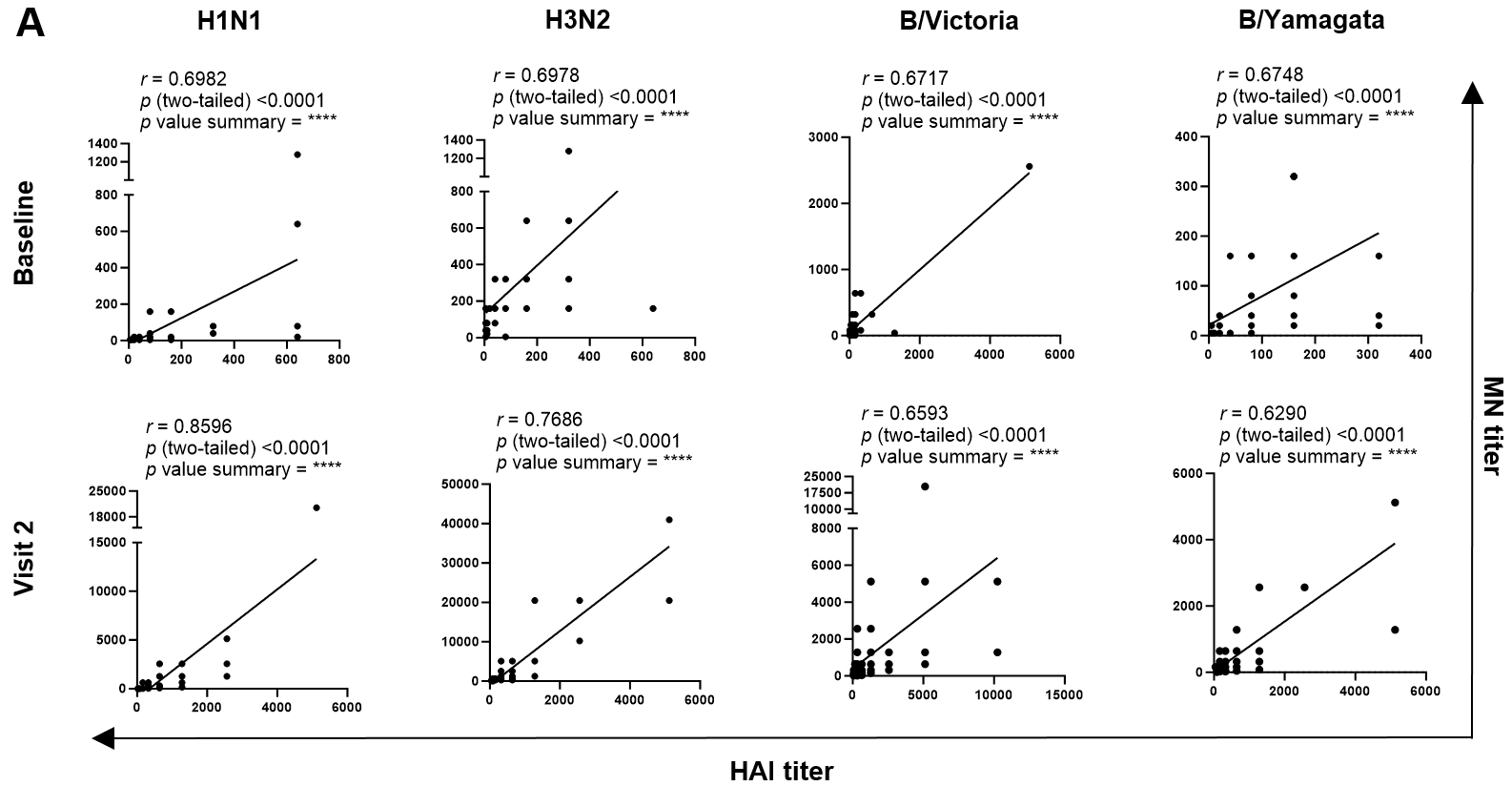


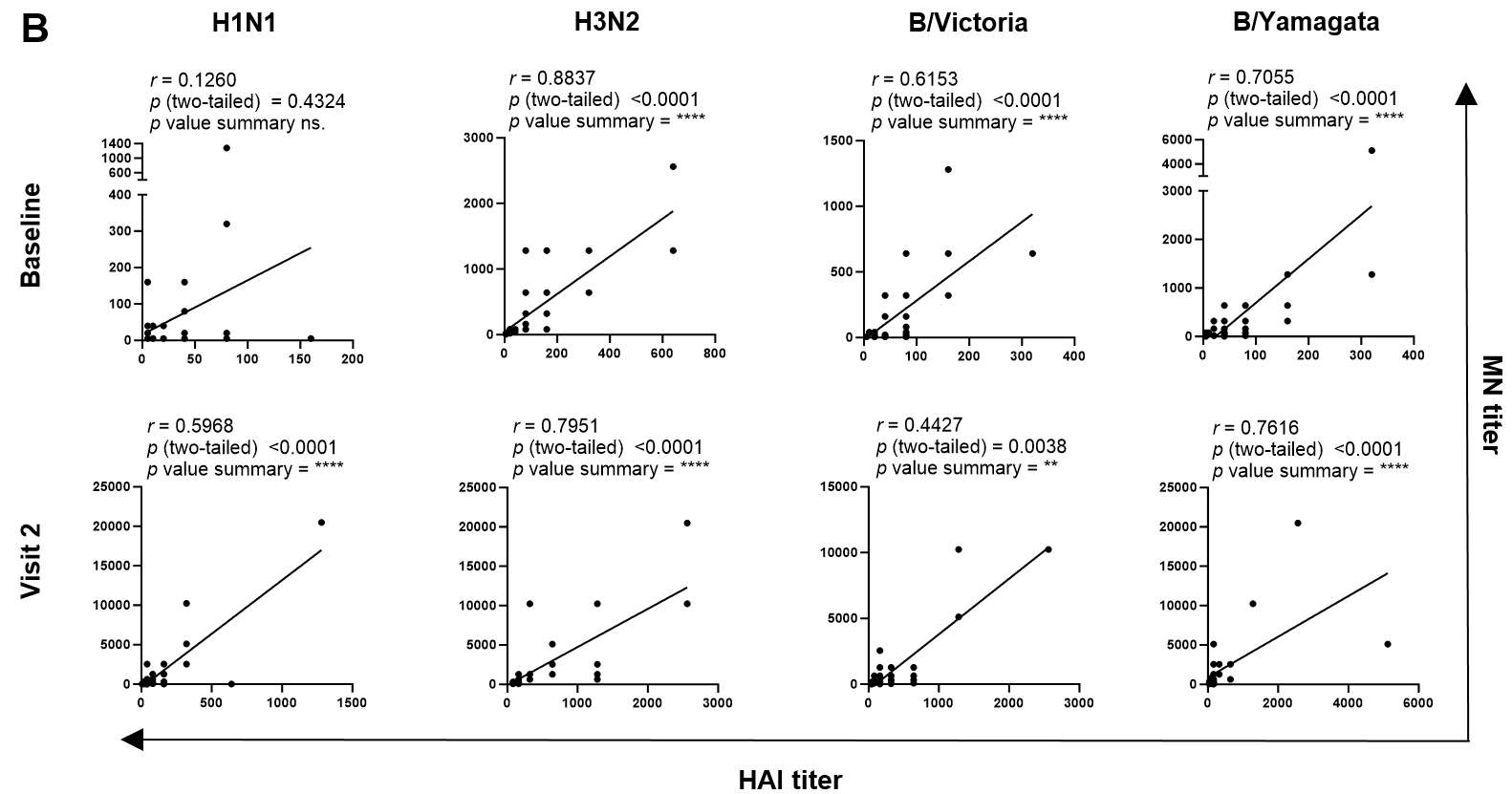


**Supplementary Figure 4: Correlation analysis between HAI and MN titers. (A**) 1^st^ season. **(B)** 2^nd^ season. Correlation analysis between HAI and MN titers with Spearman correlation coefficient r and statistical significance.

**Supplementary Figure 5**


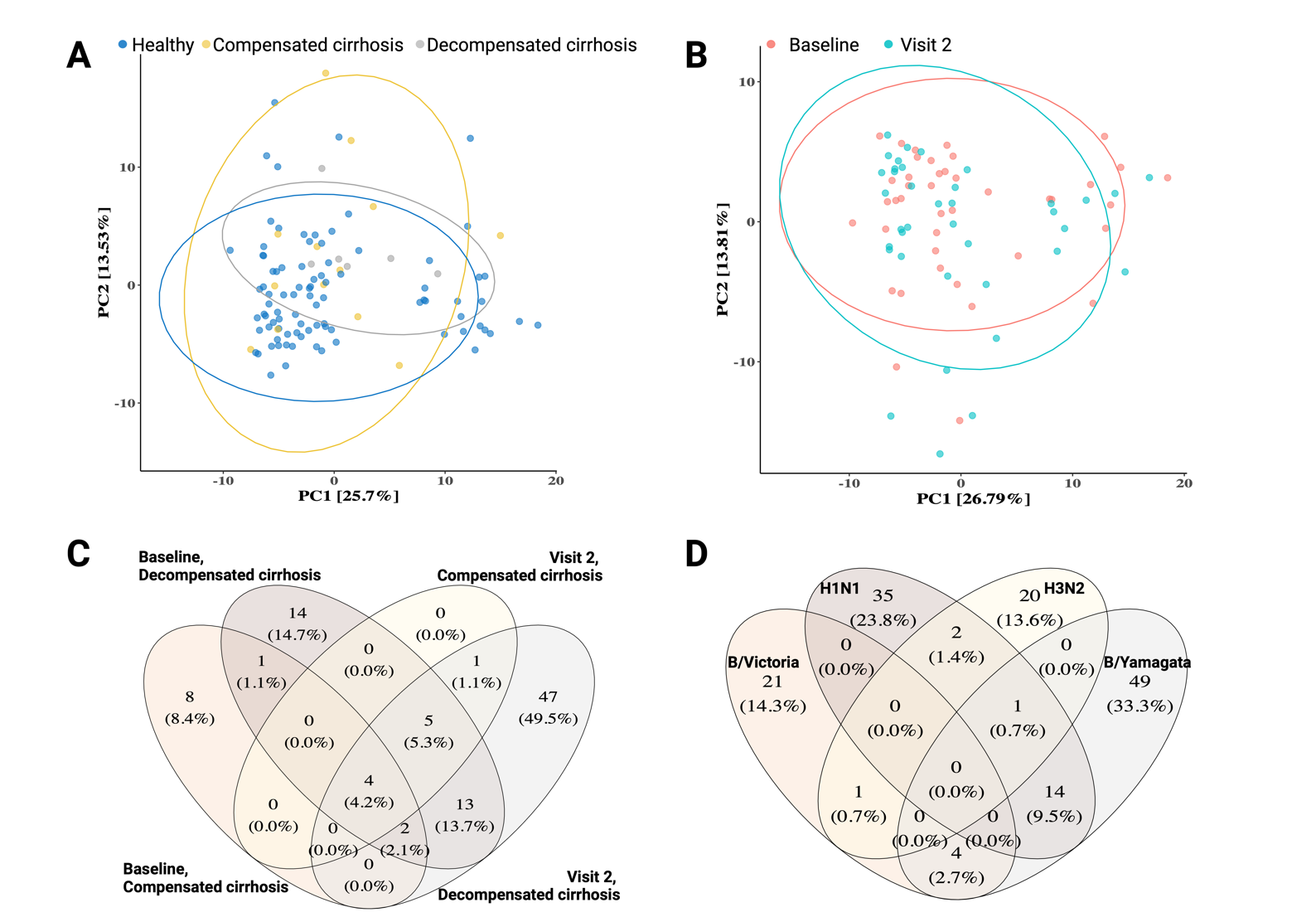


**Supplementary Figure 5: Circulating metabolite profiles of vaccinees samples obtained in the 2^nd^ season. (A+B)** The PCA plots represent the metabolite profiles between cirrhotic patients and healthy subjects, and between pre- and post-vaccination. **(C)** The number of metabolites that were significantly associated with cirrhotic conditions compared to healthy subjects at baseline and visit 2 after adjusting for age and sex. P-adj <0.05 **(D)** The number of metabolites at baseline that were significantly associated to post-vaccination (visit 2) HAI titer against each antigen separately after correcting for age, sex, and disease condition. P-value <0.05.

**Supplementary Figure 6**


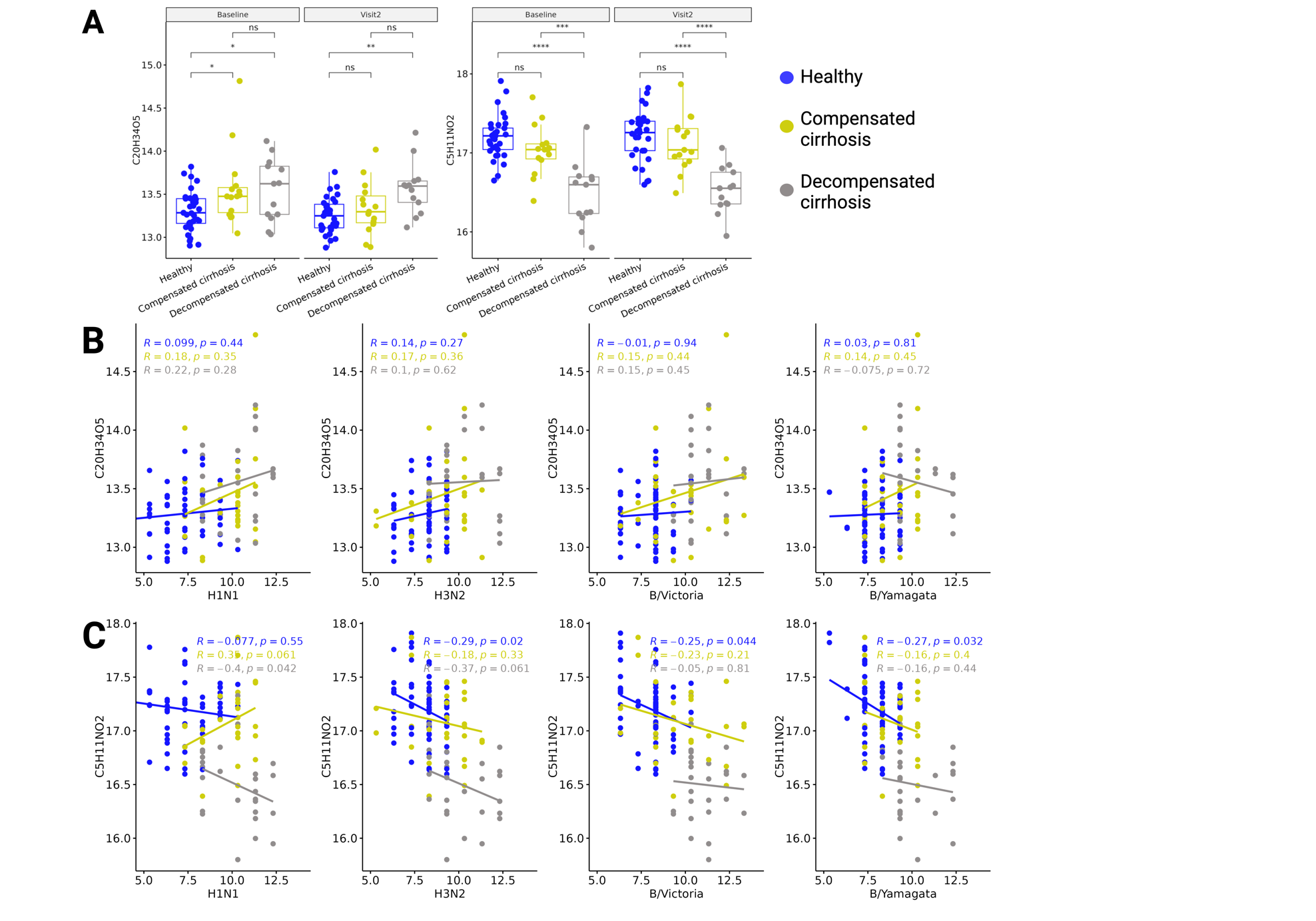


**Supplementary Figure 6: C20H34O5 and C5H11NO2 profiles from samples obtained in the 1^st^ season. (A)** The circulated metabolite abundance (the mass spectrometry peak intensity) at baseline and post-vaccination (visit 2). Box plots depict all subjects with median and quartiles for each health condition. Two-way t-test was applied for statistical significance. **(B+C)** The correlation between the circulating metabolite abundance (the mass spectrometry peak intensity) and the post-vaccination HAI titer at visit 2 (log2 value) against each antigen separately. Scatter plot depict all subjects with the linear regression line, Spearman correlation coefficient R and p-value between baseline metabolite level and post-vaccination.

**Supplementary Figure 7**


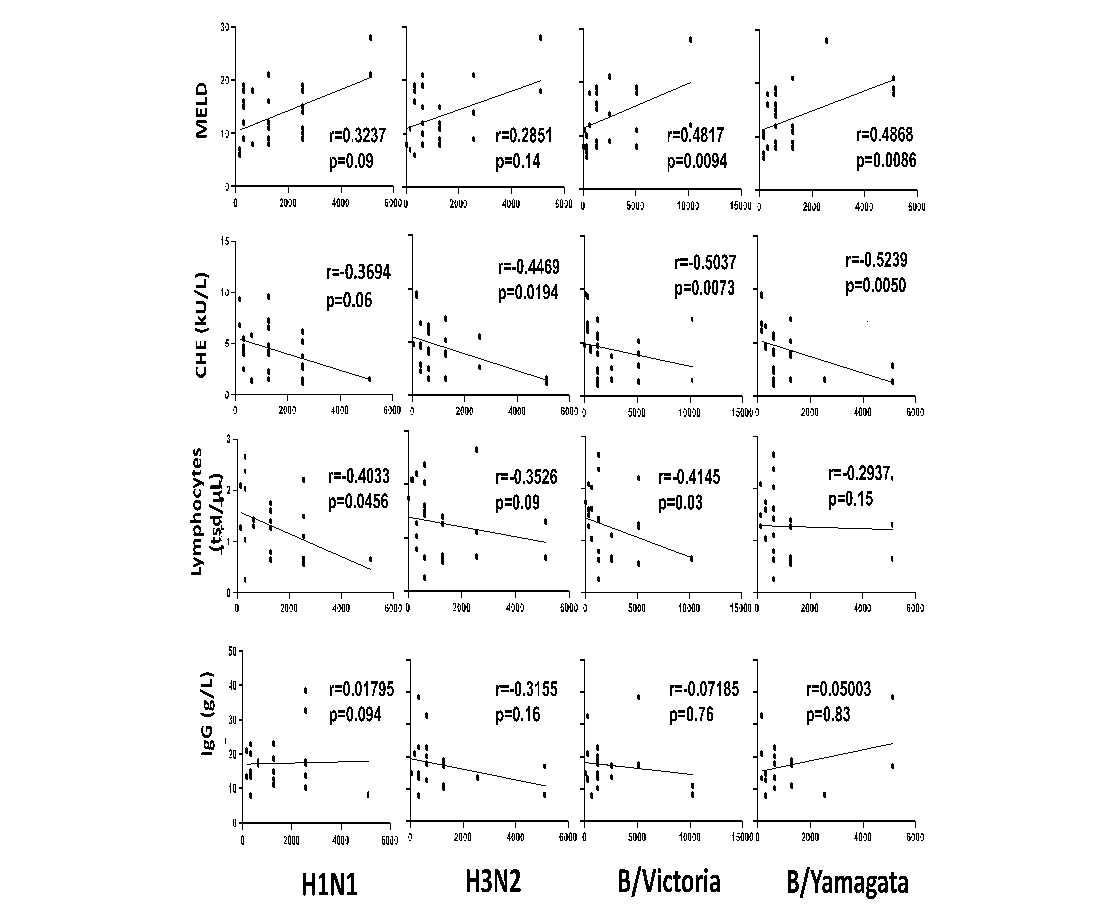


**Supplementary Figure 7: Correlation analysis between post-vaccination HAI titers in samples obtained in the 1^st^ season with routine blood markers and the MELD Score.** Parameters representing indicators for the liver function (MELD score and cholinesterase level) as well as parameters of the immune system (IgG level and lymphocyte counts) were selected for the analysis. Numbers represent the Spearman correlation coefficient r and statistical significance between clinical baseline parameters and post-vaccination (visit 2) HAI titers of cirrhotic patients.

**Supplementary Table 1**

| **Antigen** | **Fluorochrome** | **Clone** | **Company** | **Dilution** | **Panel** |
| --- | --- | --- | --- | --- | --- |
| CD3 | BUV661 | UCHT1 | BD | 1:100 | T/B-cell |
| CD4 | BUV737 | SK3 | BD | 1:800 | T-cell |
| CD8 | BUV395 | HIT8a | BD | 1:400 | T-cell |
| CCR6 | APC | G034E3 | BioLegend | 1:100 | T-cell |
| CXCR3 | BV510 | G025H7 | BioLegend | 1:100 | T-cell |
| CXCR5 | PE-Cy7 | J252D4 | BioLegend | 1:300 | T-cell |
| ICOS (CD278) | BUV615 | DX29 | BD | 1:100 | T-cell |
| IFNγ | A700 | 4S.B3 | BioLegend | 1:200 | T-cell |
| TNFα | BV750 | MAb11 | BD | 1:100 | T-cell |
| IL-2 | PB | MQ1-17H12 | BioLegend | 1:100 | T-cell |
| CD19 | APC-Cy7 | SJ25C1 | BD | 1:100 | B-cell |
| CD20 | PE-Cy7 | 2H7 | eBioscience | 1:400 | B-cell |
| CD27 | PerCP-Cy 5.5 | M-T271 | BD | 1:50 | B-cell |
| CD38 | PE-Cy5 | HIT2 | BLD | 1:100 | B-cell |
| CD138 | BUV563 | MI15 | BD | 1:50 | B-cell |
| DCM | UV | - | Invitrogen | 1:1000 | T/B cell |

**Supplementary Table 1: Antibodies used for flow cytometry analysis.**

**Supplementary Table 2**

|  |  | **1^st^ season** | | **2^nd^ season** | |
| --- | --- | --- | --- | --- | --- |
|  | **Study group** | **BL** | **V2** | **BL** | **V2** |
| HAI assay | Healthy | 33 | 33 | 33 | 33 |
|  | Compensated cirrhosis | 15 | 15 | 5 | 5 |
|  | Decompensated cirrhosis | 13 | 13 | 3 | 3 |
| MN assay | Healthy | 33 | 33 | 33 | 33 |
|  | Compensated cirrhosis | 15 | 15 | 5 | 5 |
|  | Decompensated cirrhosis | 13 | 13 | 3 | 3 |
| Flow cytometry  T cell populations | Healthy | 33 | 32 | 33 | 33 |
|  | Compensated cirrhosis | 15 | 15 | 5 | 5 |
|  | Decompensated cirrhosis | 13 | 13 | 3 | 3 |
| Flow cytometry  B cell populations | Healthy | 32 | 33 | 33 | 33 |
|  | Compensated cirrhosis | 15 | 15 | 5 | 5 |
|  | Decompensated cirrhosis | 13 | 13 | 3 | 3 |
| Cytokine profile | Healthy | 33 | 33 | 33 | 33 |
|  | Compensated cirrhosis | 15 | 15 | 4 | 4 |
|  | Decompensated cirrhosis | 13 | 13 | 3 | 3 |
| Circulating metabolite profile | Healthy | 33 | 31 | 33 | 30 |
|  | Compensated cirrhosis | 15 | 15 | 5 | 5 |
|  | Decompensated cirrhosis | 13 | 13 | 3 | 3 |

**Supplementary Table 2: Number of samples derived from vaccinees for each performed assay in the 1^st^ and 2^nd^ season.**

**Supplementary Table 3**

|  | 1^st^ season | 2^nd^ season |
| --- | --- | --- |
|  | **Cirrhotic patients (N=28)** | **Cirrhotic patients (N=8)** |
| Ascites at timepoint of vaccination | 9 (32.1%) | 3 (37.5%) |
| ascites °I | 2 (7.1%) | 0 (0%) |
| ascites °II | 1 (3.6%) | 1 (12.5%) |
| ascites °III | 6 (21.4%) | 2 (25%) |
| Previous hydropic decompensations | 6 (21.4%) | 4 (50%) |
| Esophageal varices | 19 (67.9%) | 6 (75%) |
| no information about variceal status | 3 (10.7%) | 1 (12.5%) |
| no varices | 6 (21.4%) | 1 (12.5%) |
| Varices °I | 9 (32.1%) | 0 (0%) |
| Varices °II | 7 (25%) | 5 (62.5%) |
| Varices °III | 3 (10.7%) | 1 (12.5%) |
| ß-blocker therapy | 13 (46.4%) | 6 (75%) |
| Previous varical bleeding | 5 (17.9%) | 1 (12.5%) |
| Hepatorenal syndrome | 4 (14.3%) | 1 (12.5%) |
| Hepatopulmonal syndrome | 1 (3.6%) | 0 (0%) |
| Recurrent encephalopathy | 4 (14.3%) | 1 (12.5%) |
| TIPS insertion | 5 (17.9%) | 3 (37.5%) |

TIPS (Transjugular intrahepatic portosystemic shunt).

**Supplementary Table 3: Cirrhosis-associated complications in cirrhotic study participants.**

**Supplementary Table 4**

|  | **Cirrhotic patients** | | |
| --- | --- | --- | --- |
|  | **1^st^ season (N=28)** | **2^nd^ season (N=8)** | **Total (N=36)** |
| Male sex | 22 (78.57 %) | 7 (87.5%) | 29 (80.5%) |
| Age (years) | 52 (27-71) | 59.5 (53-79) | 53.5 (27–79) |
| BMI (kg/m2) | 28 (17.2–50.8) | 27.22 (20.02-42.02) | 27.7 (17.2- 50.8) |
| Previous flu vaccinated (number of subjects)* | 10/15 (66.6%) | 1/8 (12.5%) | 11/23 (47.8%) |
| Multiple study participation | - | 1 (12.5%) | 1 (2.8%) |
| Number of smoking subjects | 6 (21.4%) | 2 (25%) | 8 (22.2%) |
| **Laboratory parameters** | | | |
| White cell count (tsd/µl) | 5.1 (2.1-9.1) | 5.8 (2-11.3) | 5.2 (2.0-11.3) |
| Lymphocytes (tsd/µl) | 1.3 (0.25-2.65) | 0.96 (0.4-2.73) | 1.27 (0.25-2.73) |
| Platelets (tsd/µl) | 86 (35-273) | 145 (39-225) | 96 (35-273) |
| INR | 1.2 (0.95-1.73) | 1.2 (0.9-1.51) | 1.2 (0.9-1.73) |
| Creatinine (mmol/l) | 84.5 (53-242) | 84 (76-160) | 84.5 (53-242) |
| Sodium (mmol/l) | 138.5 (123-144) | 136.5 (130-141) | 138 (123-144) |
| Albumin (g/l) | 38.5 (26-51) | 38.5 (33-45) | 38.5 (26-51) |
| Bilirubin (mmol/l) | 17.5 (5-143) | 13.5 (7-42) | 17 (5-143) |
| AST (U/l) | 42 (21-168) | 39.5 (22-109) | 42 (21-168) |
| ALT (U/l) | 40.5 (13-221) | 25 (11-134) | 38.5 (11-221) |
| IgG (g/l) | 17.5 (7.9-38.5) | 13.82 (12.78-15.44) | 15.14 (7.9-38.5) |
| IgM (g/l) | 1.3 (0.3-6.4) | 0.76 (0.39-2.36) | 1.2 (0.3-6.4) |
| HbA1c % | 5.25 (4-11) | 4.9 (4.6-5.8) | 5.2 (4-11) |
| **Child-Pugh Score** | 5.5 (5-10) | 6 (5-8) | 6 (5-10) |
| A (5-6 points) | 15 (53.57 %) | 5 (62.5%) | 20 (55.56%) |
| B (7-9 points) | 11 (39.29%) | 3 (37.5%) | 14 (38.89%) |
| C (< 10 points) | 2 (7.14%) | 0 (0%) | 2 (5.56%) |
| MELD | 10.5 (6-21) | 10 (5-16) | 10 (5-21) |
| CLIF-C AD ^7^ | 44 (33-61) | 48.5 (38-58) | 44.8 (33.1-61.0) |
| **Etiology of cirrhosis** | | | |
| HBV +/- HDV | 14 (50 %) | 1 (12.5%) | 15 (41.7%) |
| HCV | 6 (21.4%) | 1 (12.5%) | 7 (19.4%) |
| ALD | 2 (7.1%) | 3 (37.5%) | 5 (13.8%) |
| MASH | 3 (10.7%) | 1 (12.5%) | 4 (11.1%) |
| Unknown/ other | 3 (10.7%) | 2 (25%) | 5 (13.8%) |
| **Active chronic viral hepatitis at timepoint of vaccination** | | | |
| Total | 16 (57.1%) | 1 (12.5%) | 17 (47.2%) |
| HBV +/- HDV | 13 (46.4 %) | 1 (12.5 %) | 14 (38.9 %) |
| HCV | 2 (7.1%) | 0 (0%) | 2 (5.55%) |

*= if data were not available/ not known/ no vaccination card available previous vaccination was count as “no”

Continuous variables are expressed in median (min-max), categorial variables are expressed as total numbers (% of the respective subgroup).

INR (international normalized ratio); AST (aspartate aminotransferase); ALT (alanine aminotransferase); IgG (immunoglobuline G); IgM (immunoglobuline M); MELD (model of end stage liver disease); CLIF-C AD (CLIF consortium organ failure score); HBV (hepatitis B virus); HDV (hepatitis D virus); ALD (Alcohol-related Liver Disease); MASH (Metabolic Dysfunction-associated Steatohepatitis); HCV (hepatitis C virus)

**Supplementary Table 4: Baseline parameters of cirrhotic subjects.**

**Supplementary Table 5**

|  | **Healthy subjects** | | |
| --- | --- | --- | --- |
|  | **1^st^ season (N=33)** | **2^nd^ season (N=33)** | **Total (N=66)** |
| Male sex | 17 (51.5%) | 14 (42.4%) | 31 (46.96 %) |
| Age (years) | 40 (25-65) | 45 (21-81) | 43 (21-81) |
| BMI (kg/m2) | 22.4 (19.3-27.5) | 23.1 (18.5-31.3) | 22.76 (18.5-31.3) |
| Previous flu vaccinated (number of subjects) | 22/28 (75.9%) | 25/28 (89.3%) | 47/56 (83.9%) |
| Multiple study participation | - | 14 (42.4%) | 14 (21.2%) |
| Number of smoking subjects | 1 (2.9%) | 1 (3%) | 2 (3%) |
| **Laboratory parameters** | | | |
| White cell count (tsd/µl) | 5.9 (4-10.2) | 5.5 (4-10.7) | 5.8 (4-10.7) |
| Lymphocytes (tsd/µl) | 2.05 (1.09-3.47) | 1.68 (0.84-2.58) | 1.81 (0.84-3.47) |
| Platelets (tsd/µl) | 244.5 (192-386) | 248 (158-398) | 247.0 (158-398) |
| INR | 0.89 (0.83-0.99) | 0.91 (0.81-1.0) | 0.9 (0.81-1.0) |
| Creatinine (mmol/l) | 77 (57-108) | 77 (7-96) | 77 (7-108) |
| Sodium (mmol/l) | 140 (135-144) | 139 (134-144) | 139 (134-144) |
| Albumin (g/l) | 46.5 (41-56) | 45 (39-54) | 46 (39-56) |
| Bilirubin (mmol/l) | 9 (3-32) | 9 (3-24) | 9 (3-32) |
| AST (U/l) | 24.5 (13-53) | 20 (15-32) | 21 (13-53) |
| ALT (U/l) | 21.5 (13-79) | 20 (11-41) | 20 (11-79) |
| IgG (g/l) | 11.46 (5.26-13.79) | 10.63 (5.57-13.99) | 11.05 (5.26-13.99) |
| IgM (g/l) | 0.9 (0.25-1.85) | 0.96 (0.26-19.1) | 0.93 (0.25-19.1) |
| HbA1c % | 5.1 (4.6-7.4) | 5.3 (4.8-6.1) | 5.25 (4.6-7.4) |

Continuous variables are expressed in median (min-max), categorial variables are expressed as total numbers (% of the respective subgroup).

INR (international normalized ratio); AST (aspartate aminotransferase); ALT (alanine aminotransferase); IgG (immunoglobuline G); IgM (immunoglobuline M).

**Supplementary Table 5: Baseline parameters of healthy subjects.**

**Supplementary Table 6**

| **Comorbidities** | **Healthy subjects (N=33)** | **Cirrhotic patients (N=28)** |
| --- | --- | --- |
| Bronchial asthma | 2 (6.1%) | 0 (0%) |
| Arterial hypertension | 1 (3%) | 6 (21.4%) |
| Psoriasis vulgaris | 1 (3%) | 2 (7.1%) |
| Allergic rhinitis | 3 (9.1%) | 0 (0%) |
| Gastroesophageal reflux disease/ gastritis | 0 (0%) | 6 (21.4%) |
| Crohn disease | 0 (0%) | 1 (3.6%) |
| SLD | 0 (0%) | 1 (3.6%) |
| Migraine | 2 (6.1%) | 0 (0%) |
| Hyperlipidemia | 1 (3%) | 1 (3.6%) |
| Hypothyroidism | 2 (6.1%) | 2 (7.1%) |
| Celiac disease | 1 (3%) | 0 (0%) |
| Neurodermatitis | 1 (3%) | 0 (0%) |
| Central retinal vein occlusion | 0 (0%) | 1 (3.6%) |
| (status post) Abdominal hernia | 0 (0%) | 2 (7.1%) |
| Chronic renal failure | 0 (0%) | 2 (7.1%) |
| Prostate hyperplasia | 0 (0%) | 2 (7.1%) |
| Obesity | 0 (0%) | 3 (12.5%) |
| status post IVDA | 0 (0%) | 1 (3.6%) |
| Polyps of the colon | 0 (0%) | 1 (3.6%) |
| Chronic heart failure | 0 (0%) | 1 (3.6%) |
| Diabetes mellitus type II | 0 (0%) | 4 (15.6%) |
| Chronic pancreatitis | 0 (0%) | 1 (3.6%) |
| Endometrial hyperplasia | 0 (0%) | 1 (3.6%) |
| status post Ovariectomy | 0 (0%) | 1 (3.6%) |
| Hepatocellular carcinoma | 0 (0%) | 1 (3.6%) |
| status post Apoplexy | 0 (0%) | 0 (0%) |
| status post Appendectomy | 0 (0%) | 3 (9.4%) |
| status post HCV infection | 0 (0%) | 1 (3.6%) |
| Uterus myomatosus | 0 (0%) | 1 (3.6%) |
| Sleep apnoea syndrome | 0 (0%) | 1 (3.6%) |
| status post Testicle carcinoma | 0 (0%) | 1 (3.6%) |
| Ulcerative colitis | 0 (0%) | 1 (3.6%) |
| Congenital pulmonary valve vitium | 0 (0%) | 1 (3.6%) |
| Diverticulosis | 0 (0%) | 1 (3.6%) |
| Sarcopenia | 0 (0%) | 0 (0%) |

SLD (steatotic liver disease); IVDA (Intravenous drug abuser); HCV (hepatitis C virus).

**Supplementary Table 6: Comorbidities of study participants of the 1^st^ season.**

**Supplementary Table 7**

| **Comorbidities** | **Healthy subjects (N=33)** | **Cirrhotic patients (N=8)** |
| --- | --- | --- |
| Bronchial asthma | 3 (9%) | 0 (0%) |
| Arterial hypertension | 5 (15.2%) | 4 (50%) |
| Pulmonary hypertension | 0 (0%) | 1 (12.5%) |
| Psoriasis vulgaris | 1 (3%) | 1 (12.5%) |
| Allergic rhinitis | 1 (3%) | 0 (0%) |
| Gastroesophageal reflux disease/ gastritis | 0 (0%) | 0 (0%) |
| Crohn disease | 0 (0%) | 0 (0%) |
| SLD | 1 (3%) | 0 (0%) |
| Migraine | 2 (6%) | 0 (0%) |
| Hyperlipidemia | 0 (0%) | 0 (0%) |
| Hypothyroidism | 0 (0%) | 2 (25%) |
| Celiac disease | 0 (0%) | 0 (0%) |
| Neurodermatitis | 0 (0%) | 0 (0%) |
| Central retinal vein occlusion | 0 (0%) | 0 (0%) |
| (status post) Abdominal hernia | 0 (0%) | 2 (25%) |
| Chronic renal failure | 0 (0%) | 2 (25%) |
| Prostate hyperplasia | 0 (0%) | 1 (12.5%) |
| Obesity | 1 (3%) | 3 (37.5%) |
| status post IVDA | 0 (0%) | 0 (0%) |
| Polyps of the colon | 0 (0%) | 1 (12.5%) |
| Chronic heart failure | 0 (0%) | 1 (12.5%) |
| Diabetes mellitus type II | 0 (0%) | 1 (12.5%) |
| Chronic pancreatitis | 0 (0%) | 1 (12.5%) |
| Endometrial hyperplasia | 0 (0%) | 0 (0%) |
| status post Ovariectomy | 0 (0%) | 0 (0%) |
| Hepatocellular carcinoma (single lesion) | 0 (0%) | 2 (25%) |
| status post Apoplexy | 0 (0%) | 0 (0%) |
| status post Appendectomy | 0 (0%) | 0 (0%) |
| status post HCV infection | 0 (0%) | 2 (25%) |
| Uterus myomatosus | 0 (0%) | 0 (0%) |
| Sleep apnea syndrome | 0 (0%) | 0 (0%) |
| status post Testicle carcinoma | 0 (0%) | 0 (0%) |
| Ulcerative colitis | 0 (0%) | 0 (0%) |
| Congenital pulmonary valve vitium | 0 (0%) | 0 (0%) |
| Diverticulosis | 0 (0%) | 0 (0%) |
| Sarcopenia | 0 (0%) | 0 (0%) |
| status post Thyroidectomy | 0 (0%) | 1 (12.5%) |
| Depression | 0 (0%) | 1 (12.5%) |
| Pancreatic cystic lession | 0 (0%) | 1 (12.5%) |
| status post Myocardial infarction | 0 (0%) | 1 (12.5%) |
| Anaemia | 0 (0%) | 2 (25%) |
| status post Cholecystectomy | 0 (0%) | 2 (25%) |
| Chronic sinusitis | 0 (0%) | 0 (0%) |
| Polyps of the gall bladder | 0 (0%) | 1 (12.5%) |
| Caroli syndrome with recurrent cholangitis | 0 (0%) | 1 (12.5%) |
| Hay fever | 2 (6%) | 0 (0%) |
| Korsakow syndrome | 0 (0%) | 1 (12.5%) |
| Coxarthrosis | 1 (3%) | 0 (0%) |
| Polymyalgia rheumatica | 1 (3%) | 0 (0%) |
| Acid reflux | 1 (3%) | 0 (0%) |

SLD (steatotic liver disease); IVDA (Intravenous drug abuser); HCV (hepatitis C virus).

**Supplementary Table 7: Comorbidities of study participants of the 2^nd^ season.**

**Supplementary Table 8**

|  |  | **Seroprotection *** | | **Seroconversion**  **(mean ± SD)** | **p-value** |
| --- | --- | --- | --- | --- | --- |
|  | **Study**  **group** | **Baseline** | **Visit 2** |  |  |
| A/H1N1 | Healthy | 20 (60.6%) | 28 (84.8%) | 12.1 (±31.9) | - |
|  | Compensated  cirrhosis | 8 (53.3%) | 15 (100%) | 82.3 (±96.3) | <0.0001 |
|  | Decompensated  cirrhosis | 7 (53.8%) | 13 (100%) | 121.5 (±144.8) | <0.0001 |
| A/H3N2 | Healthy | 29 (87.9%) | 33 (100%) | 6.6 (±11.8) | - |
|  | Compensated  cirrhosis | 8 (53.3%) | 15 (100%) | 77.1 (±132.0) | 0.0014 |
|  | Decompensated  cirrhosis | 9 (69.2%) | 13 (100%) | 83.3 (±138.0) | 0.0004 |
| B/Victoria | Healthy | 28 (84.8%) | 33 (100%) | 5.9 (±11.1) | - |
|  | Compensated  cirrhosis | 11 (73.3%) | 15 (100%) | 46.6 (±51.9) | <0.0001 |
|  | Decompensated  cirrhosis | 8 (61.5%) | 13 (100%) | 177.7 (±294.6) | <0.0001 |
| B/Yamagata | Healthy | 32 (97.0%) | 33 (100%) | 4.5 (±10.9) | - |
|  | Compensated  cirrhosis | 9 (60.0%) | 15 (100%) | 36.3 (±49.7) | <0.0001 |
|  | Decompensated  cirrhosis | 10 (76.9%) | 13 (100%) | 42.2 (±50.0) | <0.0001 |

* HAI antibody titers of ≥40 were considered as seroprotective**.**

**Supplementary Table 8: Seroprotection and seroconversion rate in the 1^st^ season**. HAI antibody titers of vaccinees in the 1^st^ season were used to evaluate the seroprotection rate and the seroconversion rate. Data are represented as n (%) and mean ± SD of fold-change, respectively. Significance comparison between seroconversion rate of healthy group and cirrhosis group is based on the Mann-Whitney test.

**Supplementary Table 9**

|  |  | **Seroprotection *** | | **Seroconversion**  **(mean ± SD)** | **p-value** |
| --- | --- | --- | --- | --- | --- |
|  | Study  group | **Baseline** | **Visit 2** |  |  |
| A/H1N1 | Healthy | 11 (33.3%) | 27 (81.8%) | 7.3 (±12.5) | - |
|  | Compensated  cirrhosis | 3 (60%) | 5 (100%) | 42.4 (±53.9) | 0.0218 |
|  | Decompensated  cirrhosis | 2 (66.7%) | 3 (100%) | 16.7 (±15.0) | 0.1608 |
| A/H3N2 | Healthy | 28 (84.8%) | 33 (100%) | 7.4 (±10.1) | - |
|  | Compensated  cirrhosis | 3 (60%) | 5 (100%) | 61.6 (±108.8) | 0.0133 |
|  | Decompensated  cirrhosis | 2 (66.7%) | 3 (100%) | 48.7 (±69.1) | 0.1576 |
| B/Victoria | Healthy | 26 (78.8%) | 33 (100%) | 7.4 (±15.0) | - |
|  | Compensated  cirrhosis | 3 (60%) | 5 (100%) | 48.0 (±50.3) | 0.0005 |
|  | Decompensated  cirrhosis | 1 (33.3%) | 3 (100%) | 54.7 (±65.0) | 0.0319 |
| B/Yamagata | Healthy | 24 (72.7%) | 33 (100%) | 4.9 (±7.7) | - |
|  | Compensated  cirrhosis | 4 (80%) | 5 (100%) | 26.0 (±23.8) | 0.0071 |
|  | Decompensated  cirrhosis | 1 (33.3%) | 3 (100%) | 55.0 (±63.5) | 0.0035 |

* HAI antibody titers of ≥40 were considered as seroprotective**.**

**Supplementary Table 9: Seroprotection and Seroconversion rate in the 2^nd^ season**. HAI antibody titers of vaccinees in the 2^nd^ season were used to evaluate the seroprotection rate and the seroconversion rate. Data are represented as n (%) and mean ± SD of fold-change, respectively. Significance comparison between seroconversion rate of healthy group and cirrhosis group is based on the Mann-Whitney test.

**Supplementary Table 10**

|  | | **Baseline** | | **Visit 2** | |
| --- | --- | --- | --- | --- | --- |
|  | **Study**  **group** | **HAI** | **MN** | **HAI** | **MN** |
| A/H1N1 | Healthy | 42.6  (25.9-70.0) | 13.7  (8.0-23.6) | 127.0  (80.9-199.2) | 40.0  (19.8-80.8) |
|  | Compensated  cirrhosis | 30.3  (14.1-65.1) | 6.6  (4.3-10.2) | 884.4  (525.6-1488) | 351.0  (115.3-1068) |
|  | Decompensated  cirrhosis | 24.7  (9.3-65.5) | 7.7  (5.1-11.5) | 1214  (633.3-2325) | 675.1  (120.6-3779) |
| A/H3N2 | Healthy | 70.5  (50.2-99.0) | 210.2  (148.6-297.5) | 248.7  (193.3- 320.0) | 823.5  (570.1-1189) |
|  | Compensated  cirrhosis | 28.9  (12.2-68.1) | 91.9  (49.5-170.6) | 557.2  (305.3-1017) | 1689  (684.1-4170) |
|  | Decompensated  cirrhosis | 44.5  (15.5-127.4) | 198.0  (108.5-361.5) | 1151  (595.3- 2224) | 3525  (1170-10623) |
| B/Victoria | Healthy | 80.0  (54.2-118.0) | 45.4  (26.9-76.6) | 248.7  (190.5-324.7) | 114.3  (70.4-185.6) |
|  | Compensated  cirrhosis | 40.0  (15.8- 101.3) | 41.9  (15.3-115.0) | 844.5  (393.0- 1815) | 702.0  (380.2-1296) |
|  | Decompensated  cirrhosis | 40.0  (12.8- 124.4) | 29.1  (8.0-105.2) | 2068  (1300- 3292) | 1584  (792.9-3166) |
| B/Yamagata | Healthy | 107.3  (84.5- 136.2) | 53.7  (31.8-90.7) | 248.7  (196.3- 315.1) | 114.3  (72.2-181.2) |
|  | Compensated  cirrhosis | 33.2  (18.4-59.9) | 11.5  (6.1-21.7) | 485.0  (301.1- 781.3) | 231.5  (100.8-532.0) |
|  | Decompensated  cirrhosis | 55.0  (24.8- 122.0) | 23.5  (9.3-59.1) | 1151  (638.7- 2073) | 881.3  (430.0-1806) |

**Supplementary Table 10: Determination of influenza-specific HAI and MN GMTs in samples obtained during the** 1^st^ **season**. Serum samples of vaccinees from pre-vaccination (baseline) and post-vaccination (visit 2) in the 1^st^ season were tested against each individual vaccine antigen and virus strain in HAI assay and MN assay, respectively. Data show GMTs with lower and upper 95% CI of each study group.

**Supplementary Table 11**

|  | | **Baseline** | | **Visit 2** | |
| --- | --- | --- | --- | --- | --- |
|  | **Study**  **group** | **HAI** | **MN** | **HAI** | **MN** |
| A/H1N1 | Healthy | 14.6  (9.5-22.4) | 11.3  (7.0-18.5) | 52.6  (36.9-74.8) | 238.5  (121.6-467.5) |
|  | Compensated  cirrhosis | 17.4  (4.2-71.6) | 10.0  (1.5-68.5) | 320.0  (174.1-588.1) | 1114  (102.3-12139) |
|  | Decompensated  cirrhosis | 25.2  (0.7-907.9) | 20.0  (0.05-7789) | 254.0  (7.0-9151) | 1016  (0.4-2e+06) |
| A/H3N2 | Healthy | 94.6  (63.0-142.3) | 238.5  (139.6-407.5) | 333.7  (242.6-459.1) | 1253  (762.3-2061) |
|  | Compensated  cirrhosis | 23.0  (3.9-134.0) | 34.8  (6.7-182.3) | 485.0  (114.9-2047) | 735.2  (357.8-1510) |
|  | Decompensated  cirrhosis | 50.4  (6.9-368.0) | 80.0  (14.3-447.6) | 806.3  (22.4-2e+04) | 2032  (26.7-1e+05) |
| B/Victoria | Healthy | 44.4  (31.5-62.6) | 59.6  (31.7-112.2) | 150.2  (115.5-195.5) | 166.9  (96.3-289.0) |
|  | Compensated  cirrhosis | 30.3  (3.8-240.9) | 23.0  (1.7-312.6) | 844.5  (316.5-2253) | 1940  (267.5-14071) |
|  | Decompensated  cirrhosis | 50.4  (0.9-2688) | 40.0  (0.08-19874) | 1280  (228.8-7162) | 3225  (89.5-1e+05) |
| B/Yamagata | Healthy | 45.4  (31.0-66.4) | 163.4  (87.2-306.2) | 121.8  (94.6-156.8) | 429.4  (278.0-663.1) |
|  | Compensated  cirrhosis | 60.6  (22.7-161.8) | 91.9  (15.8-536.2) | 970.1  (229.8-4095) | 3880  (1052-14313) |
|  | Decompensated  cirrhosis | 31.8  (4.3-231.8) | 63.5  (4.6-881.1) | 1016  (139.1-7419) | 5120  (259.4-1e+05) |

**Supplementary Table 11: Determination of influenza-specific HAI and MN GMTs in samples obtained during the 2^nd^ season**. Serum samples of vaccinees from pre-vaccination (baseline) and post-vaccination (visit 2) in the 2^nd^ season were tested against each individual vaccine antigen and virus strain in HAI assay and MN assay, respectively. Data show GMTs with lower and upper 95% CI of each study group.

**Supplementary Table 12**

| **Pathway** | **Source** | **Member input overlap** | **Member input formula** | **Size** | **Effective size** | **p-value** | **q-value** |
| --- | --- | --- | --- | --- | --- | --- | --- |
| Arachidonic acid metabolism - Homo sapiens (human) | KEGG | C14809; C14782; C14811; C05959; C00639; C14814; C13809 | C20H34O5; C20H34O5; C20H34O5; C20H34O5; C20H34O5; C20H34O5; C20H34O5 | 75 | 25 | 4.54e-05 | 0.0015 |
| Synthesis of Hepoxilins (HX) and Trioxilins (TrX) | Reactome | C14811; C04843 | C20H34O5; C13H19N4O12P | 10 | 3 | 0.00548 | 0.0853 |
| Valine, leucine and isoleucine biosynthesis - Homo sapiens (human) | KEGG | C04411; C04236; C02504; C00183 | C7H12O5;  C7H10O5;  C7H12O5;  C5H11NO2 | 23 | 20 | 0.00915 | 0.0853 |

**Supplementary Table 12: Pathway analysis outcome for significant metabolite in the 1^st^ season (Figure 6).** Metabolite formulas were converted to KEGG ID, and then used to perform the pathway analysis. The pathway name, source, member input overlap, size, effective size, p-value, and q-value were derived from the ConsensusPathDB-human website. Only significant pathways (p-value <0.05) are shown in the table.

**Supplementary Table 13**

| **Parameter** | **High responder all**  **[N; median (min-max)]** | **Low/non-responder all**  **[N; median (min-max)]** | **p-value** |
| --- | --- | --- | --- |
| Male sex | 36; 25/36 (69.4%) | 25; 14/25 (56%) | 0.4165 |
| Age (years) | 36; 46.50 (25.00-71.00) | 25; 44.00 (31.00-65.00) | 0.4593 |
| BMI (kg/m2) | 30; 27.06 (17.16-50.78) | 9; 24.62 (20.55-27.46) | 0.1494 |
| Previous flu vaccinated (number of subjects) | 36; 13/36 (36.1%) | 20; 19/20 (95%) | <0.0001 |
| white cell count (tsd/µl) | 36; 5.2 (2.10-9.10) | 24; 5.9 (2.90-10.20) | 0.0593 |
| Lymphocytes (tsd/µl) | 33; 1.49 (0.25-3.26) | 24; 2.05 (0.63-3.47) | 0.0021 |
| Platelets | 36; 127.0 (35.0-386.0) | 24; 242.5 (42.0-342.0) | 0.0003 |
| INR | 35; 1.19 (0.85-1.63) | 24; 0.89 (0.83-1.73) | <0.0001 |
| Creatinine (mmol/l) | 36; 81.5 (53.0-242.0) | 24; 79.5 (59.0-114.0) | 0.6666 |
| Sodium (mmol/l) | 36; 139.0 (123.0-144.0) | 24; 139.5 (135.0-144.0) | 0.3732 |
| Albumin (g/l) | 35; 43.00 (26.00-51.00) | 24; 46.00 (29.00-56.00) | 0.0022 |
| Bilirubin (mmol/l) | 34; 13.00 (3.00-71.00) | 24; 11.00 (4.00-39.00) | 0.0440 |
| AST (U/l) | 35; 38.00 (13.00-168.0) | 24; 24.50 (13.00-41.00) | <0.0001 |
| ALT (U/l) | 35; 30.00 (13.00-221.0) | 24; 20.50 (13.00-66.00) | 0.0301 |
| IgG (g/l) | 30; 13.35 (5.26-38.51) | 23; 11.76 (8.20-21.02) | 0.0623 |
| IgM (g/l) | 30; 1.105 (0.30-3.26) | 23; 0.9100 (0.25-1.85) | 0.1433 |
| HbA1c % | 28; 5.100 (4.00-11.00) | 23; 5.300 (4.60-7.40) | 0.5308 |
| CHE (kU/l) | 34; 5.315 (1.09-12.97) | 24; 8.405 (1.53-13.52) | <0.0001 |
| LDL (mmol/l) | 29; 2.18 (0.66-109.0) | 23; 3.24 (2.19-5.080) | <0.0001 |

INR (international normalized ratio); AST (aspartate aminotransferase); ALT (alanine aminotransferase); IgG (immunoglobuline G); IgM (immunoglobuline M).

**Supplementary Table 13: Differences in baseline parameters between high responders vs. low/non-responders participating in the 1^st^ season.** Vaccinees, who were identified as triple or tetra responders, were considered as high responders, whereas low/non-responders include non, single and double responders.
