# Supplementary material for: Particularly strong immune response to influenza vaccination in patients with decompensated liver cirrhosis linked to systemic inflammation
Source: Front Immunol. 2026 Apr 22;17:1734093. doi: 10.3389/fimmu.2026.1734093 (PMC13143674; doi:10.3389/fimmu.2026.1734093)
Supplement: Supplementary file 2 [file DataSheet2.pdf]

The International Society for the Advancement of Cytometry (ISAC) has highlighted the importance of including comprehensive methodological information to ensure data reproducibility and reliability. In line with this, Frontiers in Immunology now requires authors to submit a checklist for manuscripts that involve flow or mass cytometry. This checklist helps standardize the reporting process, improving the quality and transparency of published data. By doing so, we support scientific progress, making it easier for other researchers to replicate and validate experiments.

This form should be submitted with any manuscripts using flow or mass cytometry.

Sample/specimen/material description

☐ Total blood

☑ PBMCs

☐ Organ digests

Other____

Did the samples suffer any treatment before or after incubation with the antibodies?

☑ Drug Monensin, Brefeldin A

☑ Cell permeabilization Cytofix/Cytoperm

☑ Dye Viability dye (DCM)

☐ Propidium iodine

☐ Not applicable

Other ____

Instrument and antibodies

Name of the Cytometer BD FACSymphony A5

Antibodies and targets	Fluorochrome/ Metal	Catalog number/Company
anti-CD3	BUV661	Cat. 612964/BD Biosciences
anti-CD4	BUV737	Cat. 612749/BD Biosciences

anti-CD8	BUV395	Cat. 740303/BD Biosciences
anti-CCR6	APC	Cat. 353416/BioLegend
anti-CXCR3	BV510	Cat. 353726/BioLegend
anti-CXCR5	PE-Cy7	Cat. 356924/BioLegend
anti-ICOS (CD278)	BUV615	Cat. 751092/BD Biosciences
anti-IFNy	A700	Cat. 502520/BioLegend
anti-TNFa	BV750	Cat. 566359/BD Biosciences
anti-IL-2	PB	Cat. 500324/BioLegend
anti-CD19	APC-Cy7	Cat. 557791/BD Biosciences
anti-CD20	PE-Cy7	Cat. 25-0209-42/eBioscience
anti-CD27	PerCP-Cy5.5	Cat. 560612/BD Biosciences
anti-CD38	PE-Cy5	Cat. 303507/BioLegend
anti-CD138	BUV563	Cat. 748351/BD Biosciences

Data analyses

1. Name of the software___FlowJo (BD Biosciences)___________
2. Reference gating strategy in the manuscript or supplementary material

Gating strategy in (eg Figure X) ___Supplementary Figure 1_________
